# Candidate Genes for the High-Altitude Adaptations of Two Mountain Pine Taxa

**DOI:** 10.3390/ijms22073477

**Published:** 2021-03-27

**Authors:** Julia Zaborowska, Bartosz Łabiszak, Annika Perry, Stephen Cavers, Witold Wachowiak

**Affiliations:** 1 Institute of Environmental Biology, Adam Mickiewicz University in Poznań, Uniwersytetu Poznańskiego 6, 61-614 Poznań, Poland; bartosz.labiszak@amu.edu.pl (B.Ł.); witold.wachowiak@amu.edu.pl (W.W.); 2 Centre for Ecology and Hydrology-Edinburgh Site, Bush Estate, Penicuik, Midlothian EH26 0QB, UK; annt@ceh.ac.uk (A.P.); scav@ceh.ac.uk (S.C.); 3 Institute of Dendrology, Polish Academy of Sciences, Parkowa 5, 62-035 Kórnik, Poland

**Keywords:** candidate genes, high-altitude adaptations, mountain pines, outlier loci, *Pinus*, SNP genotyping array

## Abstract

Mountain plants, challenged by vegetation time contractions and dynamic changes in environmental conditions, developed adaptations that help them to balance their growth, reproduction, survival, and regeneration. However, knowledge regarding the genetic basis of species adaptation to higher altitudes remain scarce for most plant species. Here, we attempted to identify such corresponding genomic regions of high evolutionary importance in two closely related European pines, *Pinus mugo* and *P. uncinata*, contrasting them with a reference lowland relative—*P. sylvestris*. We genotyped 438 samples at thousands of single nucleotide polymorphism (SNP) markers, tested their genetic differentiation and population structure followed by outlier detection and gene ontology annotations. Markers clearly differentiated the species and uncovered patterns of population structure in two of them. In *P. uncinata* three Pyrenean sites were grouped together, while two outlying populations constituted a separate cluster. In *P. sylvestris*, Spanish population appeared distinct from the remaining four European sites. Between mountain pines and the reference species, 35 candidate genes for altitude-dependent selection were identified, including such encoding proteins responsible for photosynthesis, photorespiration and cell redox homeostasis, regulation of transcription, and mRNA processing. In comparison between two mountain pines, 75 outlier SNPs were found in proteins involved mainly in the gene expression and metabolism.

## 1. Introduction

The sessile lifestyle of plants forces them to develop adaptations, enabling them to quickly, reversibly, and often enduringly cope with environmental changes or colonize new niches to avoid competition. Because photoperiod and temperature are important environmental factors for plants, long-lived trees must make trade-offs between survival, regeneration, development, and reproduction, fitting annual growth cycles in response to seasonal length variations [1]. For alpine species, in addition to usually strong contraction of the vegetation time, the most serious stressors include reduced atmospheric pressure, photooxidative stress, lowered temperatures, and demanding substrates [2,3,4]. In addition, plants are frequently exposed to strong winds, torrential rains, and avalanches, particularly affecting trees above the forest line. Mountain trees not only need to balance growth and dormancy periods through phenological adaptations but must also evolve morphological, anatomical, and physiological modifications to adjust their metabolisms or withstand mechanical damage [5,6,7,8]. Therefore, we were interested in exploring the genetic basis of the adaptations of such trees to high altitudes. Many tree species, including the pines on which the survey was conducted, maintain high fertility and enforce strong selection between recruited individuals, which facilitates adaptation to new environments. At the same time, these trees are characterized by large effective population sizes, considerable dispersion potential of pollen and seeds and outcrossing, all of which contribute to rapid homogenization of gene pools within their populations, thereby hindering the consolidation of beneficial novelties [1]. In mountain habitats, the populations are usually insular and are subjected to rapid changes in environmental factors along the elevation gradient. These features affect gene exchange and promote the diversification of stands but tend to limit variation within them [9,10,11]. Biometric and quantitative genetic studies have helped to elucidate patterns of differentiation; however, knowledge remains scarce regarding the genetic basis of plants adaptation to higher altitudes and patterns of differentiation at genomic regions of high adaptive importance.

The genus *Pinus* in the Pinaceae family of conifers has a widespread distribution, mostly in the Northern Hemisphere, with nearly 100 species being described [12], including taxa associated with mountain habitats. In Europe, two well-defined high-altitude sister taxa from the *Pinus mugo* complex are known: the dwarf mountain pine (*Pinus mugo* (Turra)) and the Pyrenean mountain pine (*P. uncinata* (Ramond)). The dwarf mountain pine is a shrubby species that creates dense carpets on the ground, occasionally reaching 3 m in height. This species inhabits stands above tree lines, up to about 2700 m [13,14]. The Pyrenean mountain pine is a typical erect tree up to 25 m in height, which itself establishes the upper limits of forest and trees between altitudes of 1400 and 2700 m. Both species grow on rocks and debris, and their ecological niches appear to be comparable; however, to the best of our knowledge, this similarity was never specifically tested. The taxa meet in a wide area of the Western Alps, where both populations of exclusively one species and mixed stands, often with ongoing hybridization, are observed [15,16,17], except that their current ranges are essentially disjointed. The major populations of dwarf pine occupy subalpine regions of the Alps, Sudetes, and Carpathians and southern mountain chains in Romania and the Balkan Peninsula, as well as several smaller, remote populations, as in Abruzzo in Italy [13,18]. The Pyrenean pine, aside from stands in the Western Alps and the core populations in the Pyrenees, inhabits higher parts of the Massif Central, Ligurian Apennines, Jura and Vosges, as well as several more isolated areas spread over the Iberian Peninsula [19,20].

Although these two pines share a common history, have similar genomic backgrounds, and occupy comparable environments that demand the same specific adaptations, they are differentiated by a number of phenotypic and genetic characteristics. Biometric studies showed that in addition to their different growth forms, these two species differ in various cone and needle characteristics [15,18,21]. Under a common garden experiment, they exhibited notable differences in the pace of growth of young trees and in important phenological properties related to the timing of bud set and bud burst [22]. The species possess the same numbers of chromosomes, but ongoing divergence has been found in their karyotypes [23]. More detailed molecular studies, although underlining common history and genetic background, also indicated some significant changes that appeared in the genomes of these pines [24,25,26], such as fixed variants at mitochondrial DNA [17].

As the species overlap to a certain extent across altitude, temperature, and rainfall ranges, we might expect patterns of adaptive phenotypic variation to be similar for both. Generally, variation between species may be a consequence of adaptive changes, including selection for species-specific or high-frequency divergent alleles accumulated over the time of independent evolution, but this variation may partially result from mutation load, recombination, and demography. To date, attempts to identify potential drivers of the adaptation of these trees to mountain habits or to investigate their mutual dissimilarity have been notably limited and have focused on a small number of genetic regions. This small number of studies reflects the overall challenge in pine research related to their longevity and long generation time, which make experimental investigations impractical. Molecular studies focused on identifying potential loci under selection are also demanding, considering the large and complex genomes of pines, that hamper the acquisition of a reliable whole genome reference assembly (over 20 gigabases, rich in retrotransposons and repetitive sequences [27,28,29]). Although some effort has been made to explore the patterns of neutral genetic variation between populations at plastid, nuclear, and mitochondrial markers [16,17,30], considerable research remains to be performed. To date, studies investigating genetic variation at the coding regions of these taxa have included sets of 12 and 79 candidate genes, respectively [25,31].

In the present study, we attempted to broaden our knowledge regarding the patterns of polymorphisms and divergence in two subalpine taxa to identify subjects of selection potentially responsible for their adaptations and speciation. We analyzed these two species because their evolutionary history has not been fully elucidated, and because they are both potentially threatened by ongoing habitat loss [14,20,32]. We intentionally focused on interspecies comparisons, contrasting not only the two mountain pines with each other, but also with respect to the Scots pine (*P. sylvestris* L.). The Scots pine is a species with vast but mostly lowland distribution and is the closest living relative of both mountain pines from which they diverged approximately 5 million years ago [33,34]. We conducted genome-wide analysis using a genotyping array recently developed for these pines [35]. This new Axiom_PineGAP chip (Affymetrix, Thermo Fisher Scientific, Waltham, MA, USA) was composed of 49,829 single nucleotide polymorphism (SNP) markers derived from the reference transcriptome [26], some resequenced genes and other markers known from these and other pine genomes, which appears to be especially useful for comparative evolutionary investigations. To elucidate the drivers of adaptation to higher altitudes and identify regions responsible for the further differentiation of these species, most likely caused by adjustments to local conditions, we first verified application of the SNP array to distinguish the species and subsequently evaluated levels of polymorphism and divergence along with the population structure of analyzed stands. Finally, using several approaches, we investigated outlier SNPs that strongly differ between investigated species, assessed the functions of the source genes and compared the results with previous findings concerning these taxa and other high-altitude or similarly phenotypically differentiated taxa.

## 2. Results

### 2.1. Genetic Diversity

**Figure 1:**
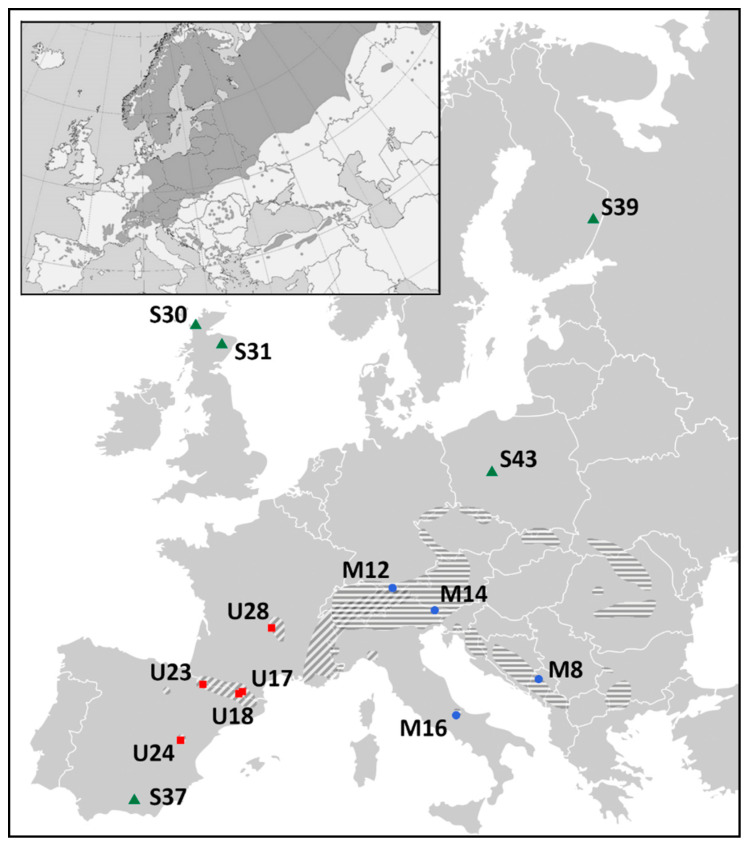
Localization of 14 studied populations with indication of species distributions. Dwarf pine—blue dots and horizontal shading, Pyrenean pine—red squares and diagonal shading, Scots pine—green triangles and dark shading on attached miniature presenting species’ European range. The mountain pines’ distribution map was created by the authors based on the Empty Political Map of Europe iso3166-1, downloaded from Wikimedia Commons (https://commons.wikimedia.org, accessed 4 October 2018; [36]), and the information on species’ ranges taken from [19]. The Scots pines’ distribution map has been obtained by courtesy of the EUFORGEN (EUFORGEN 2009, www.euforgen.org, accessed 22 September 2009; [37]), and adapted.

After quality filtering of SNPs and individuals following our criteria of genomic origin and potential linkage of markers, frequency of alleles (MAF), percentage of individuals properly genotyped per marker, and percentage of SNPs genotyped per individual, the data set consisted of 6003 polymorphisms and 438 samples: 79 dwarf pines, 182 Pyrenean pines, and 177 Scots pines, derived from 14 populations spread across Europe (Figure 1, Supplementary Table S1).

The populations varied considerably in terms of the percentage of polymorphic loci. This index ranged from 61.1% to 91.3%, exhibiting a mean over the 14 populations that was equal to 80.4%, but higher averages within species reached above 90% (Table 1). The majority of the variants were shared, and none of the variants was fixed in a species while being absent from others. The taxa differed considerably in the numbers of unshared alleles (Supplementary Figure S1), which in most cases were species- and not population-specific (Table 1). The measures of mean within group distances (*d*), which in the case of Scots pine’s populations did not exceed 1000, ranged between 1193.4 (M14) and 1437.1 (U18) in the stands of other two species. The observed heterozygosity (*Ho*) was generally higher than the unbiased expected heterozygosity (*uHe*), resulting in multiple negative values of fixation indices (*F*) in populations. Two heterozygosity estimates did not differ notably in terms of mean values between species; however, in all cases, except for *uHe* between mountain pines, the differences were highly statistically significant (*p* < 0.001). In populations, *Ho* exhibited the lowest levels in Scots pine (from 0.160), while populations of the two mountain pines exhibited higher values (up to 0.338; Table 1). An analogous pattern of low variability in Scots pine and larger variability in the remaining taxa was observed in the case of the expected heterozygosity (*uHe*); however, in the latter two species, the statistics decreased in relation to *Ho*, while the Scots pine’s average increased (Table 1). The fixation index (*F*) calculated on the whole dataset appeared to be positive (0.081), which was also observed in Scots pine (0.067) and its two populations; nevertheless, the majority of investigated stands exhibited greater diversity than was expected.

### 2.2. Differentiation and Grouping of Populations

To validate the robustness of our marker set in terms of discrimination power among analyzed species and populations, and to utilize it for the reconstruction of relationships among them, we assessed our data using a series of distance, differentiation, and clustering methods.

First, distances based on numbers of differences within and among groups were calculated and compared. The overall mean distance (d = 1459.6) was higher than that observed between individuals of Scots (d = 1003.5) or dwarf mountain pine (d = 1308.7) and only slightly larger than observed among individuals of Pyrenean pine (d = 1426.8); however, it was markedly lower than the distances between taxa (Table 2). The largest gap among species (*d_xy_* = 1761.9) was observed between Scots and dwarf pines; the second largest gap, which was considerably smaller, was between Scots and Pyrenean pines with *d_xy_* = 1552.3, while the two mountain species were separated by 1523.5 nucleotides of differences on average. A similar pattern was also reflected in the results obtained from respective comparisons of population pairs (Supplementary Table S2a,b), with exceptions only being observed in cases of populations U28 and U24, which showed a closer relation with Scots pines’ populations than to populations of dwarf pine and the majority of populations from their own taxon. Based on these distances, a UPGMA tree was constructed (Supplementary Figure S2a), which depicted precise separation of Scots pine and dwarf pine with a less clear position of the third species. Populations of Pyrenean pine appeared to be split into two clusters, with one, consisting of populations U17, U18, and U23 (all from the Pyrenees) being set at the base of the dwarf pine branch, and the other—populations U24 and U28—being set at the base of the Scots pine part. On the dendrogram constructed without Scots pine, dwarf, and Pyrenean pines occupied separate branches (Supplementary Figure S2b).

Analysis of molecular variance (AMOVA) with populations hierarchically grouped within their respective species demonstrated that the majority of variance present in our data segregated inside populations (73.28%), and the variation between them was considerably lower (5.43%) than that among taxa (21.28%); all these results were statistically significant (*p* < 0.001). In the AMOVA with no imposed structure, 22.51% of the variation differentiated taxa and 77.49% segregated within them. The overall *Fst* fixation index values in these two versions of the test were equal to 0.2672 and 0.2251, respectively. More specific calculations of differentiation indices (Table 2, Supplementary Table S2), supported the pattern demonstrated by distance analysis. The most differentiated of all interspecific comparisons was the dwarf and Scots pine pair (*Fst* = 0.3612), and taxa from the *P. mugo* complex reached a considerably lower level of *Fst* = 0.1061 (or 0.1510 when population structure was included). Comparisons between individual populations, where *Fst* values ranged between 0.015 (M8 vs. M12) and 0.451 (M14 vs. S37), generally confirmed previous findings. One exception was found in the case of Pyrenean pine population U28, which exhibited a closer relationship with most Scots pine’s stands than with the Alpine dwarf pine provenance M14. Moreover, most of the populations in this species showed genetic similarity to dwarf pine population M8, often exhibiting higher similarity than to other populations in their own taxon. All the *Fst* values between taxa and among populations were significant (*p* < 0.001).

**Figure 2:**
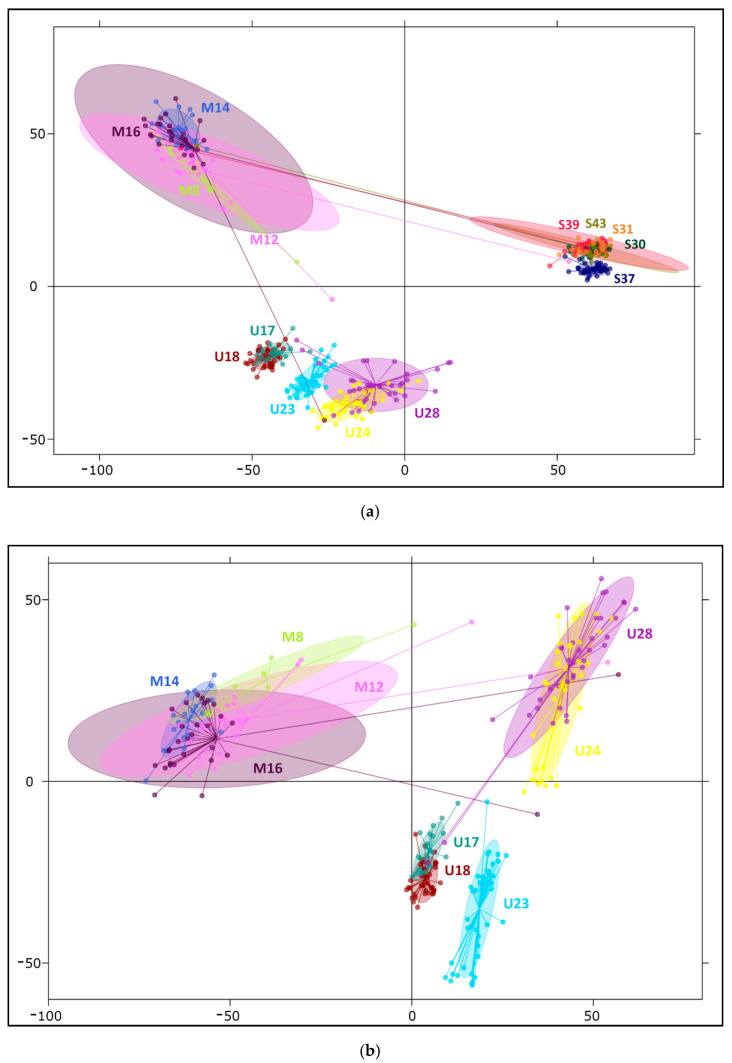
Principal component analysis (PCA) revealing relationships between and within the studied pine species. (**a**) Division of the three investigated taxa, first and second coordinates (horizontal and vertical axes, respectively) correspond to 12.3% and 3.5% of variation; (**b**) separation of two mountain species with 6.6% of variation explained by the first and 3.4% by the second component. Ellipses indicate 95% confidence intervals.

Among clustering analyses, two multivariate methods were implemented. To determine how our SNPs discriminate populations of studied taxa, principal coordinate analysis (PCoA) was conducted, and principal component analysis (PCA) was subsequently employed to characterize variation within populations and to elucidate individual genotypes. In comparison between all 14 investigated populations, the three first axes of PCoA explained 58.8%, 12.6%, and 5.7% of the original variation, respectively, while in PCA, considering variation among individuals, these values decreased to 12.3%, 3.5%, and 2.0% (Figure 2a and Supplementary Figure S3a), confirming that a greater part of the diversity segregates within populations. These results confirm the distinction of three gene pools correlating with the studied species. Generally, horizontal axes separated dwarf pine from Scots pine, and Pyrenean pine was distant from them along the second axis, remaining somewhat closer to the other mountain taxon. The PCA plot (Figure 2a) exhibited greater variability within species and populations and facilitated the identification of some outlier samples. Five individuals grouped with populations of other species, indicating their putative hybrid origin, or less likely, mixture of the samples. In multivariate inquiries focused on two mountain pines, PCA axes accounted for 6.6%, 3.4%, and 2% of variation (Figure 2b), and in PCoA, they explained 44.5%, 16.0%, and 11.6% of diversity (Supplementary Figure S3b), in both cases distinguishing first between taxa, then between Pyrenean pine stands (U17, U18, and U23 vs. U24 and U28). The second axis also demonstrated only slight distinction of the dwarf pine stand M8 in the Dinaric Alps from the rest of its populations; however, 95% confidence intervals of the dwarf pine populations remained overlapping.

To verify the hypothesis of isolation by distance (IBD), i.e., to formally determine whether genetic differences are larger between populations when geographic gaps among them increase, we performed Mantel tests of correlation between genetic and geographic distances. This analysis was needed to ensure that STRUCTURE analyses would not be affected by bias caused by data with allele frequencies varying gradually, as the model underlying this clustering method is not well suited for such scenarios. These analyses were computed for the whole set of 14 populations, as well as in two-species comparisons and within taxa. Except for intraspecies tests in all other cases, these correlations were positive and significant (Supplementary Table S3 and Figure S6a,b). We consider the three negative outcomes—weak and insignificant correlations of within-species inspections; and the fact that the positive outputs might have been biased by specific geographic distributions of studied populations and generally unequal ranges of the taxa to be sufficient to not expect any disturbances in further tests.

**Figure 3:**
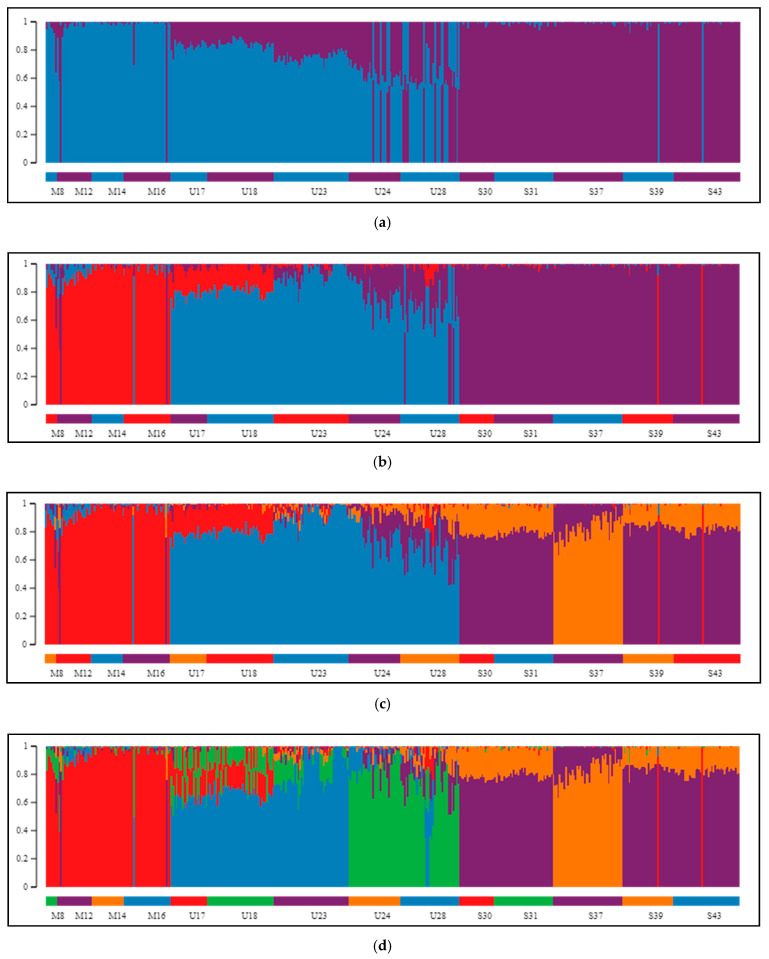

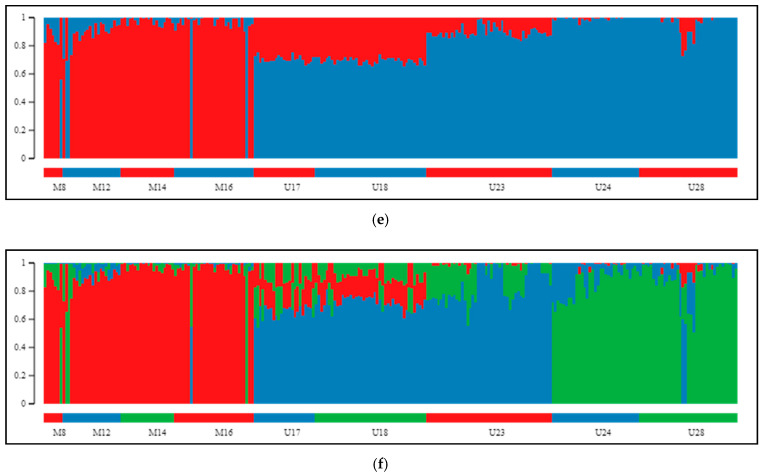
Clustering of 14 inspected pine populations, as proposed by the STRUCTURE algorithm. Separation of populations of three species into (**a**) two, (**b**) three, (**c**) four, or (**d**) five distinct groups. *K* = 2 and *K* = 5 were pointed out as the best supported clustering, depending on whether the result for *K* = 1 was under consideration. (**e**) After exclusion of Scots pine samples, *K* = 2 was indicated as the most likely assemble, closely corresponding with the two mountain taxa. (**f**) The second best supported structure of the tested *P. mugo* complex populations consisted of three groups (*K* = 3): dwarf pine specimens, Pyrenean pine representatives from Pyrenees and its accessions from isolated populations in Sierra de Gudar and Massif Central together. Scale on the left and vertical bars represent the proportion of each genome being composed of variants specific to particular clusters. Horizontal color bars at the bottom of each chart label distinct populations.

The Bayesian clustering method implemented in STRUCTURE, which was performed on our SNP data, helped to elucidate the genetic structuring of individuals. Upon two employed methods (Evanno and L(K) method) for indication of best genetically explicit grouping from the examined range of *K* values between 1 and 10, division into two groups (Figure 3a) was indicated to be optimal (Supplementary Figure S4a,b). According to STRUCTURE HARVESTER, the step between *K* = 1 and *K* = 2 was characterized by the largest change in likelihood distribution and the highest Delta*K* score. When we excluded *K* = 1 from the analysis, because this point dimmed the picture with its markedly low anticipated and obtained probability of data, the program suggested division into five clusters as the best (Figure 3b). Splitting the samples into two groups (*K* = 2, Figure 3a) validated the distinction between populations of the *P. mugo* complex and Scots pine. The division was clear in the case of the dwarf pines that had very little contribution from the second cluster, whilst among specimens of Pyrenean pine, mixing of both clusters was manifested. In these individuals, dwarf pine participation dominated based on *Q* values constituting, on average, 71% (SD = 13.5%) of their genomes. In the case of *K* = 3 (Supplementary Figure S4c), each species established its own cluster, and only in Pyrenean pine was potential gene flow from the other groups found. In populations from the Eastern Pyrenees (U17 and U18), the influence of dwarf pine was primarily observed; at the same time, populations from more remote stands (U24 and U28) displayed a considerable contribution of Scots pine in their genomes. Generally, outcomes from tests with higher *K* values (Supplementary Figure S4d–g) were characterized by the existence of a stable, coherent group of dwarf pine samples and increasing differentiation within Scots pine while still maintaining the consistency of individual populations, especially in Pyrenean pine, where three provenances showed greater variability and inner differentiation. In the exact outcome of clustering samples into five groups (Figure 3b), which was second-best based on the Delta*K* criterion, Pyrenean pine was divided into two parties, and Spanish population S37 was separated from the rest of the Scots pine’s samples.

The results of analyses focused on two mountain taxa yielded very similar patterns. First, clear discrimination between dwarf and Pyrenean pines was obtained for *K* = 2 (Figure 3c; Supplementary Figure S5a,b). The second most likely structure indicated by STRUCTURE HARVESTER was composed of three clusters, supporting strong subdivision within Pyrenean pine (Figure 3d). Models assuming the existence of more groups confirmed the substructure in Pyrenean pine populations, and only once, for *K* = 7, was differentiation in dwarf pine detected (Supplementary Figure S5c–h). Interestingly, at each grouping variant, the same few individuals were consistently observed to manifest affiliation to a cluster corresponding with a species different from the one expected, and these were exactly the same samples that already appeared inconsistent on PCA plots.

### 2.3. Outlier SNPs and Their Functional Annotation

From the pairwise comparisons between taxa, the most differentiated markers based on their frequencies and high *Fst* values were obtained. Outlier SNPs were detected with three methods—BayeScan, FLK, and FDist. We focused on the markers that were indicated by at least two methods at a threshold level of significance equal to 0.05 (depending on the analysis used, it was either *p* or *q* value). We later performed a more in-depth examination of the loci that were found simultaneously in three analyses or were significant at more stringent level (*p*/*q* values = 0.01). In total, in all the comparisons conducted between species, 120 outlier SNPs were confirmed by at least two methods at the significance level of 0.05 (Supplementary Tables S4 and S5).

**Figure 4:**
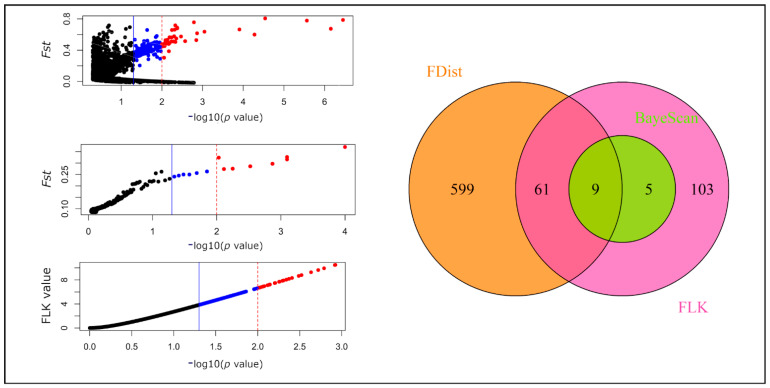
Outlier SNPs detected by three methods in comparison between populations of dwarf and Pyrenean pine. Three panels on the left represent outlier markers (significant at *p*/*q* value ≤ 0.05—blue and red points, the first values in brackets, or *p*/*q* value ≤ 0.01—red points only, the second values in brackets) revealed by FDist (207/43; **top**), BayeScan (14/8; **middle**) and FLK (178/28; **bottom**) methods. The right panel with a Venn diagram shows how the results from the less stringent level of significance in the three analyses overlap. From the output of the FDist method, only SNPs with *Fst* above 0.02 were considered significant and are colored.

When two high-altitude taxa were together compared with the Scots pine populations, 35 detected SNPs corresponded in at least two tests at a significance level of 0.05. These markers closely correlated with those found to differentiate between Pyrenean and Scots pines (29 outliers), while only one such marker was detected between the last species and dwarf pine (Supplementary Table S4). Between the two mountain species 75 highly divergent SNPs were identified (Figure 4). However, only 9 SNPs were indicated by all three methods at the same level, whereas at a more stringent threshold of 0.01 at least two methods agreed on 9 markers; eight were common to these two sets and were considered to be the most reliable outliers (Table 3 and Supplementary Table S4). No fixed SNP allele was observed in any of three taxa that would be absent from the others (Supplementary Table S5).

Each of the 120 highly divergent outliers was subjected to Gene Ontology annotation. Sequences from the reference transcriptome (contigs) on which the SNPs were called and derived from were subjected to OmixBox, where they underwent phases of BLAST search, GO mapping, gene annotation, InterProScan annotation and final merging of the results from last two steps. From the initial input 5 contigs yielded no alignment in the xblast search (additional screening of the expressed sequence tags (EST) database with NCBI blastn to identify related nucleotide sequences that could substitute the original queries proved equally inefficient). Furthermore, 15 sequences failed at the BLAST quality filtering, mapping or annotation step, leaving 100 loci that successfully passed the whole annotation process, described by 239 different GO terms in total (Supplementary Table S4). The determined gene ontologies represented each of the three general GO domains—biological processes (BP), cellular components (CC), and molecular functions (MF), and numbers of terms assigned to sequences varied strongly reaching maximum of 14 ontologies in one case.

Three comparisons among Scots and mountain pines (MU vs. S, M vs. S, and U vs. S) yielded only one marker that was supported by them all, and it was the singular marker that diverged between dwarf and Scots pines. This particular SNP was observed in a transcript (comp28590_c0_seq1) with very good xblast and annotation quality. The sequence was described as photosystem I P700 chlorophyll A apoprotein A2 (phyA), an integral component of the thylakoid membrane, which participates in electron transfer during photosynthesis. Except for that particular sequence, both alpine taxa were differentiated from Scots pine in 34 other loci, of which 29 were correctly annotated. Half of these markers (17) were also found at variance in Pyrenean pine and its lowland relative when they were analyzed independently along with 12 additional outlier loci not found in any other comparison (Supplementary Table S4).

From genes with SNPs differentiating two focal pines, 241 terms were elucidated. More of the ontologies belonged to MF (45 different terms found for 50 out of 64 efficiently annotated transcripts) and BP domains (42 terms for 48 transcripts) than were affiliated with CC (23 terms found for 38 sequences). Molecular functions were most commonly represented by the binding of organic cyclic compounds, heterocyclic compounds, or ions and by transferase, transcription regulator, and catalytic activities (Supplementary Figure S7). Among biological processes, metabolic processes (organic substances, cellular, primary, or nitrogen compound metabolic processes) and biosynthesis were most heavily represented. Gene products of the sequences with outlier SNPs identified were located mainly in organelles, the membrane, often constituting its intrinsic components, and cytoplasm. In addition to examining the most frequent ontologies, we also inspected the GO terms identified for the eight most significant outliers (Table 3), which were supported by all three methods applied for detection and, in most of the cases, were significant at *p*/*q* value of 0.01 too.

## 3. Discussion

### 3.1. Discrimination of the Species

In this study, we analyzed genetic relationships between a pair of closely related coniferous taxa—dwarf mountain pine and Pyrenean mountain pine. Studies of the adaptive variation of these species are especially important due to environmental changes and ongoing habitat loss in mountainous areas. Although these taxa face rather similar environmental pressures and share a common genetic background [25,31], they differ significantly in a number of characteristics and are widely recognized to be two separate taxa [38]. For evolutionary assessments we used a novel SNP array and the reference Scots pine to, first, determine the discrimination power of this new tool in the studied system, and to elucidate the genetic differentiation and divergence of these species. We further looked for candidate loci that could be responsible for ecological adaptation and speciation of these pines.

The number of finally used markers (6003 SNPs) appeared low compared to the number of those primary arranged on the array (almost 50 thousand). This drop was a result of a combination of factors, first of all of a medium-low conversion rate on the test set of samples (about 42%, better described in [35]). The second drop, from the 20,795 markers that passed the initial test, to the final six thousand, resulted from the worse quality of *P. mugo* genotyping in this study, probably an outcome of imbalanced mixing of species samples during array development. To keep as many individuals of this focal species as possible, we made a trade off on the number of markers, excluding those that performed the worst in *P. mugo*.

Patterns of genome-wide polymorphism at a set of nuclear SNPs in the analyzed pines showed that most variation (over 73% in AMOVA analysis) was distributed within populations. This result is in line with patterns previously found in the case of pollen transmitted chloroplast markers [16,30,39] and biparentally inherited nuclear loci [25,26,31], but is different from those observed in less mobile, seed-mediated mitochondrial genomes [17]. The levels of variation were comparable between species, and there were relatively low numbers of unshared polymorphisms detected in species and individual populations. The genetic diversity was sufficient, however, to delineate three investigated taxa and many of their populations, supporting the genetic differentiation between specimens of the Scots pine and two mountain pine species. Moreover, although some particular interspecies pairwise population comparisons deviated from the general pattern, populations within the *P. mugo* complex exhibited more common variants and their allele frequencies were more evenly distributed, as observed in distance and differentiation indices, than between any of these samples and the five Scots pine populations.

For the majority of statistical analyses, a closer relationship between Pyrenean pine and Scots pine was observed, rather than between the latter and dwarf pine, and the pattern was mostly affected by two Pyrenean pine populations. A close genetic relation between these taxa, which could have preserved more of the ancestral polymorphisms, was also observed in earlier comparative analyses of transcriptomes, phenology, and certain phenotypic traits (growth form and rate, needle characters [18,22,26]). Interestingly, notably more outliers were found in this instance in comparisons between Pyrenean and Scots pines than between the reference species and dwarf pine—1 and 35, respectively.

Our study provides a new example [40,41,42] of a successful application of a genome-wide scan for delineation of phylogenetic relationships among taxonomically challenging plant groups which, in this study, were from the *P. mugo* complex [28,38]. The two sister taxa appeared separated in multivariate analysis plots, having disjointed 95% confidence intervals in PCA tests. Definite distinction into two genetically distinct groups was also determined by Bayesian clustering of dwarf and Pyrenean pines. The species showed moderate but significant differentiation by AMOVA (*Fst* = 0.151); moreover, a larger proportion of variation was found among species (9.14%) than among populations (5.95%). Similar to previous resequencing studies of nuclear loci [25,31], no fixed differences between these pines have been demonstrated.

### 3.2. Inner Genetic Variation of Three Pine Species Populations

Our data showed only minor differentiation between dwarf pine populations. This differentiation was quite visible in constant homogeneity of dwarf pine samples on STRUCTURE charts but was also marked by the lowest values of pairwise *Fst* estimates—on average, half the size of that observed in the two other species. Only the Abruzzian site M16 emerged as an individual cluster in Bayesian clustering under *K* = 7. Earlier studies showed that trees from that population exhibit many phenotypic [18,21] and genetic [43,44] characteristics of Pyrenean pines. The present investigation on nuclear markers, however, cannot conclusively confirm the introgression hypothesis, as the population as a whole did not differ from others of its species, neither being more distant nor showing exceptional pairwise *Fst* estimates. This population also did not show signs of closer affinity to Pyrenean pine such that more than that one specimen from this stand was grouped with samples of the sister species in PCoA analysis (Figure 2) and on charts obtained from STRUCTURE (Figure 3 and Supplementary Figure S5). Therefore, the characteristics of this population most likely result from its isolation in remote location [14,45], rather than inflow from Pyrenean pine. Apart from that finding, in multivariate analyses focused on two mountain species another stand; namely, M8 from the Dinaric Alps, displayed some distinction. Nevertheless, on both variants of UPGMA trees all dwarf pine sites constituted one coherent group, which appeared to have formed in a relatively short and distant period of diversification.

More differentiated were populations of Pyrenean pine. Each of the populations displayed some symptoms of inner variation or stronger differentiation from others on STRUCTURE plots, and the highest mean within and between group distances were observed in this species and its populations. Two sets of populations, one including Pyrenean sites and the other composed of stands in Massif Central and Sierra de Gudar, were distinguished by the Bayesian method and multivariate tests. Further differentiation of these populations was observed in the Pyrenean cluster, with the northwestern population U23 being distinct from two Andorran sites, which was in keeping with prior observations of a significantly slower growth rate of pines in this region [22].

Two remote Pyrenean pine stands from France and southern Spain (U24 and U28, respectively) appeared particularly similar to each other, as they grouped together on PCA and PCoA plots, the phylogenetic dendrograms, and equally clearly in STRUCTURE analysis. These stands also exhibited the greatest diversities of individual genotypes, well expressed in measures of heterozygosity and on PCA plots. Moreover, the two populations were characterized by considerably higher similarity to Scots pine, and on the UPGMA tree with all three taxa included, they were even located at the branch specific to Scots pine. Distinctness of the two stands and their striking genetic similarity, especially in the context of significant mutual geographical distance, was observed earlier based on mitochondrial and chloroplast markers [17,30,39] and some biometric traits [18], suggesting a long evolutionary relationship and/or past introgression, especially since plausible mixing of Pyrenean and Scots pines within stands in Massif Central has already been considered [17,24].

Although Scots pine was not the focal species in our study, some observations regarding its genetic variation may be noted based on the conducted analyses. Distinct patterns of differentiation of populations within species were obtained using our two approaches. Estimates based on numbers of nucleotide differences (*d_xy_*) showed little variation among Scots pine populations compared to the other taxa, whilst conclusions based on allele frequency differentiations (*Fst*) indicated that this species is the least consistent of all. Although Scots pine appeared very consistent not only on the UPGMA tree but also on the plots obtained with multivariate analyses, STRUCTURE showed divergence of the Iberian Peninsula population (S37) that also exhibited the highest *Fst* in pairwise tests in relation to provenances of its own and of other species, which can be interpreted as a signal of its stronger spatial isolation from other species’ sites [46].

### 3.3. Candidates for Drivers of High-Altitude Adaptations in the Studied Subalpine Pines

Loci, from which the 35 outlier SNPs differentiating two mountain species from the reference taxon were derived may serve as potential targets of selection that have shaped today’s diversity of these pines. Transcripts for which gene ontology annotation was possible primarily represent proteins taking part in photosynthesis, cell redox homeostasis, photorespiration or oxidant detoxification, regulation of transcription and mRNA processing. Some of these genes encoded diverse protein-related agents (with proteolytic, kinase or methylotransferase activity), molecules participating in transport or in cortical microtubule organization. The set of identified genes corresponds to previous studies in alpine conifers and other plants, in which the existence of various adaptive strategies for reducing photodamage (a serious stressor under extended periods of high irradiance, reduced partial pressure of CO_2_, and low temperature, which are often present in high-altitude areas) has been demonstrated [47,48,49,50,51,52,53].

Transcripts containing outlier SNPs identified between dwarf and Pyrenean pines were commonly involved in metabolic and biosynthetic processes. The largest group, representing approximately one-third, was related to gene expression, and the others were responsible for the metabolism of proteins, nitrogen compounds or phosphorus, transport, and responses to stimuli. Complementarily, the prevailing molecular functions of genes were related to nucleic acid binding and transcription regulator activities. Other frequent MFs were associated with metal ion binding, protein binding and diverse catalytic activities. Additionally, one of the most significant outliers observed in this study (see comp52994_c0_seq1 in Table 3) and a homeobox domain-containing protein discovered as strongly diverged in previous interspecies studies on these pines (marker Pr1_10 in [31]) are also both involved in the regulation of transcription. This finding indicates that an important role is played by the differentiated expression of genes in the evolutionary history of the studied organisms and may partly explain the overall low differentiation of these species at the sequence level [31]. This finding may also indicate an important role played by the phenotypic plasticity mediated by transcriptional regulation in diverse responses of these pines to changes in environmental conditions [54,55]. Clearly, more detailed comparative transcriptomic studies are warranted, for which the foundation has already been established [26].

Along with the mentioned transcript encoding homeobox domain containing protein, seven other sequences containing SNPs that were highly diverged between two mountain pines, supported by three methods and significant at the 1% level, were annotated as follows: AP-4 complex subunit mu, glucan endo-1,3-beta-glucosidase 8-like, heavy metal-associated isoprenylated plant protein 36-like, strictosidine synthase-like 3 protein, phosphatidate phosphatase PAH2-like, aminodeoxychorismate synthase (chloroplastic isoform X1) and serine/threonine-protein kinase VPS15-like (isoform X1). The first molecule is an element of the adaptor protein (AP) complex and most likely functions as a vesicle coat component involved in targeting proteins from the trans-Golgi network (TGN) to the endosomal-lysosomal system [56,57], thereby participating in the regulation of intracellular transport. The second protein, recognized as glucan eno-1,3-beta-glucosidase 8, is an enzyme with hydrolase activity bound to the extracellular side of the lipid bilayer of the cell membrane. This protein catalyzes hydrolysis of beta-D-glucose units from the nonreducing ends of 1,3-beta-D-glucans, thereby releasing glucose. This protein was previously found to exhibit significant covariance with altitude and/or be related to drought stress responses in plants [58], including conifers *Pinus pinaster* [59] and *Abies alba* [60,61]. This protein was also indicated to be a climate-related candidate gene subjected to differentiating selection in two closely related Asian species of high- and low-altitude pines, namely, *Pinus hwangshanensis* and *P. massoniana* [62]. Although both species investigated in this study are restricted to mountain conditions, divergence observed between them and not between them and the reference Scots pine populations may indicate the importance of the water conditions in the speciation history of Pyrenean and dwarf pines. Another candidate was found among members of a large family of heavy metal-associated isoprenylated plant proteins (HIPPs). These metal ion-binding and transporting molecules (metallochaperones) play important roles in the development of vascular plants and their responses to environmental changes [63]. The molecules generally act in two ways, being involved in either heavy metal homeostasis and mechanisms of detoxification (in particular, tolerance to cadmium) or in the regulation of transcriptional responses to cold and drought. Strictosidine synthase-like 3 protein, as its name suggests, participates in biosynthesis of strictosidine, a precursor molecule in the monoterpenoid indole alkaloid biosynthesis pathway; however, according to Hicks et al. [64], this protein may catalyze hydrolytic reactions typical of other members of functionally diverse N6P superfamily [65], to which strictosidine synthase-like proteins belong; therefore, its role remains unclear. Another outlier-containing transcript identified in this study in comparisons of two mountain pines was described as potentially encoding phosphatidate phosphatase PAH2. This enzyme regulates cellular metabolic processes through catalysis of the dephosphorylation of phosphatidate, thereby yielding diacylglycerol. PAH2 represses the biosynthesis of phospholipids at the endoplasmic reticulum, and is involved in galactolipid synthesis, which is required for membrane remodeling [66]. This mechanism is considered an essential adaptation to cope with phosphate starvation [67], and although the chemical composition of the substrates on which the two studied plants grow was not specifically tested previously, it would be worthwhile to investigate whether the soil conditions or mycorrhizal symbionts do play important roles as ecophysiological factors determining the speciation of these pines [68,69,70,71]. Aminodeoxychorismate synthase (chloroplastic isoform X1) participates in carboxylic acid metabolic processes in plastids, where it catalyzes the biosynthesis of 4-amino-4-deoxychorismate and L-glutamate from chorismate and glutamine, part of a pathway for the biosynthesis of para-aminobenzoic acid, a precursor for folate production [72]. Folates constitute an essential family of cofactors, and are involved in virtually every aspect of plant physiology [73,74]. Disturbances in folate metabolism usually strongly inhibit growth of a plant; therefore, their altered function could be responsible for the dwarfism of the dwarf pine. The last of the eight most significant outliers was found in the transcript for a protein similar to serine/threonine-protein kinase VPS15. This enzyme is located in endosomes and is required for transport from cytoplasm to vacuole and for autophagy, while autophagy has been shown to perform numerous functions, both in normal plant growth and development and in the plant response to environmental stresses [75,76].

Considering that these species exhibit substantial distinctions in growth forms, some anatomical and morphological traits and phenology, certain genes may be responsible for the observed diversity. Good candidates could potentially be among factors regulating the development and receptivity of individuals to periodic life cycle events, such as different kinds of phytohormones. Among the uncovered outliers, only three annotated sequences matched the prediction directly; one was annotated as the ethylene-responsive transcription factor ERF112, and the other two were transcription factors acting in response to gibberellins (two isoforms of scarecrow-like protein 3 (scl3)). Although the role of the first protein is less clear, the involvement of gibberellin signaling pathways is notable. Gibberellins (GA) constitute a group of plant hormones important for the regulation of various developmental processes, such as flowering, germination, senescence of fruits and leaves, dormancy or stem elongation; therefore, genes affected by GA appear to be likely candidates for controllers behind the observed shifts in phenology and phenotypes of pines. Mutants in GA biosynthesis and downstream responses have been observed in a variety of taxa, often causing dwarfism or semi-dwarfism of plants, including alpine plants [77,78]. The role played by scl3 in enhancing dwarf phenotypes of GA signaling pathway mutants was already shown in *Arabidopsis* [79]. Alpine dwarfism is a widely observed phenomenon of plant height decrease along altitudinal gradients, often regarded as an evolutionary adaptation to extreme environmental conditions, enabling plants to take advantage of higher ambient temperatures near the ground, reduce evaporation, counteract mechanical damage from wind or snow cover, and invest more resources in reproduction [78]. It was formerly reported for other shrubby alpine pine species—*Pinus pumilla* in Asia, that their mean stem height was approximately four-fold lower at the upper distribution limit (49 cm) than at the lower limit (187 cm), and the ratio of stem height to length was also lower at the upper distribution boundary [80]. Moreover, it was demonstrated that the less developed stature of scrubs at the upper distribution limit was due to shorter stem age, more creeping stems and lower shoot elongation rates. This latter finding fits well with the known role of gibberellins in regulating the elongation of shoots [81,82], among others, in pines [83], suggesting that the scl3 protein may be involved in shrubby and prostrate development of dwarf pine, which is not observed in Pyrenean mountain pines. However, it is interesting that the same two outliers were not found in comparisons between dwarf and the reference Scots pine. The above candidate genes represent good targets for further nucleotide polymorphism and gene expression studies that may be equally important in the development of adaptive divergence among these species.

In the future, information regarding the patterns of expression of genes important in speciation history, including those demonstrated here, and of epigenetic marks could help to elucidate the evolutionary trajectories of these plants. As these differences cannot be fully explained by nucleotide variation alone, these factors are usually important drivers of phenotypic and phenological variation in long-lived, sessile organisms. A better ecological description of habitats occupied by mountain species is needed because more detailed eco-physiological findings may help to elucidate the mechanistic connection between the identified diverged sequences and the selection pressures acting on the trees. Moreover, correlation studies conducted on a larger set of populations to check if the environmental factors and genetic diversity of investigated pines vary dependently would be required, with special focus on the revealed outlier sequences. Another, well-suited test for altitude-related adaptations could incorporate association studies performed on repeated pairs of genetically homogenous populations, coming from lower versus higher altitude locations.

### 3.4. Conclusions

The nuclear loci investigated in this study showed largely homogeneous patterns of variation between the taxa; nevertheless, using a novel set of SNPs, we managed to delineate the three species. Differentiation within species appeared moderate; however, some of the patterns of population structure appeared consistent between analyses, especially the differentiation found in the Pyrenean pine population. The outlier loci possibly targeted by selection and variable between two mountain species and the reference, represented two main groups of biological functions—were involved either in oxidation-reduction processes or regulation of transcription, including transcripts engaged in the gibberellin signaling pathway, known of its important function in plant development and of its connection with dwarf phenotypes, which are important adaptations observed in high-mountain species.

Considering the longevity of pines, their predominantly outcrossing mating system with considerable pollen transport abilities and effective population sizes, our results indicate that the SNP array, providing genotyping data directly comparable between different experiments, may serve as a new diagnostic tool enhancing the discrimination power of previously limited biometric and genetic features of the species that may be useful in a variety of applications in population genetics.

## 4. Materials and Methods

### 4.1. Materials

Specimens of three closely related European pine species, i.e., dwarf mountain pine (*Pinus mugo* Turra), Pyrenean pine (*P. uncinata* Ramond), and Scots pine (*P. sylvestris* L.) were investigated. Initially, cones from five mother trees, distanced from each other by a minimum of 50 m, were collected from 14 natural populations of the taxa—four or five stands per species. The formal biometric identification of the mother trees was conducted by Prof. Krystyna Boratyńska from the Institute of Dendrology, Polish Academy of Sciences, who generously shared the material (in case of this collection, no voucher specimen has been deposited in the Institutes’ herbarium). Seedlings obtained from the open-pollinated seeds were grown under unified conditions of a common garden experiment at a glasshouse facility of the Centre for Ecology and Hydrology, Edinburgh, UK (for more details about the samples, see Wachowiak et al. [22]). From the total number of seedlings, 524 individuals were selected (141 dwarf, 201 Pyrenean and 182 Scots pines), and 22 to 47 samples per population were collected (Figure 1, Supplementary Table S1).

### 4.2. SNP Array Genotyping

DNA was extracted from needles using a DNeasy 96 Plant Kit (Qiagen, Hilden, Germany). Needles were dried on silica gel prior to extraction and DNA was quantified using a Qubit spectrophotometer (Thermo Fisher Scientific Inc., Waltham, MA, USA) to ensure a minimum standardized concentration of 35 ng/µL. We genotyped 49,829 SNPs that were used for the development of the Axiom_PineGAP SNP array (Thermo Fisher Scientific Inc., Waltham, MA, USA). Details about the array design and initial quality assessments performed on the called SNPs are described in Perry et al. [35]. Briefly, the array comprises polymorphisms from transcriptome sequencing [26] and candidate genes resequenced in previous population genetic studies of pine species [28,84,85,86]. Genotyping was performed at Edinburgh Genomics following DNA amplification, fragmentation, chip hybridization, single-base extension through DNA ligation, and signal amplification performed according to the Affymetrix Axiom Assay protocol. Genotyping was performed in 384-well format on a GeneTitan (Thermo Fisher Scientific Inc., Waltham, MA, USA) according to the manufacturer’s procedure. Genotype calls were obtained using Axiom Analysis Suite v.4.0.3.3 software as recommended by the manufacturer (Thermo Fisher Scientific Inc., Waltham, MA, USA).

From the initial set of 49,829 SNP markers designed on the array and 20,795 loci that passed basic quality tests (details available in [35]), the number of markers was further reduced by removal of (1) redundant SNPs developed from the same original sequence, (2) SNPs putatively originating from one of the organelle genomes, (3) SNPs that were monomorphic or did not pass the threshold of 1% minor allele frequency (MAF), and (4) loci with more than 15% missing data. In detail, the first step was taking one, randomly chosen, marker per contig to ensure independence between polymorphisms. Although pines are generally known for their rapid LD decay, given the limited information regarding the physical connection of the SNPs, we preferred to choose conservative approach eliminating possible effects of linkage on the analyses, even at a cost of weaker association signal. Additionally, individuals with genotypes missing at more than 15% of SNPs were also rejected. Different cut-off levels were tested (between 5% and 20%), and the chosen levels (15% per loci and 15% per individual) were considered to be the most reasonable trade-off between the quality of genotyping data and the number of excluded markers and samples. Data processing was performed using PLINK v.1.07 (http://pngu.mgh.harvard.edu/purcell/plink/, accessed on 29 August 2019; [87]), PGDSpider v.2.1.1.5 (http://www.cmpg.unibe.ch/software/PGDSpider/, accessed on 30 June 2017; [88]), and R v.3.6.1 (https://cran.r-project.org/bin/windows/base/old/3.6.1/, accessed on 22 July 2019; [89,90]).

### 4.3. Analysis of Genetic Diversity, Differentiation, and Population Grouping

We calculated several statistics of genetic diversity of the SNP dataset to assess the overall level of variation within the whole sample and on the species and population levels. Within each analyzed combination of samples, we checked (1) percentage of polymorphic sites (*%P*), (2) number of private polymorphisms (*Sp*), (3) mean within group distance (*d*), (4) observed heterozygosity (*Ho*), (5) unbiased expected heterozygosity (*uHe*) taking into account sample sizes, and (6) fixation index (*F*). Calculations of *%P*, *Sp*, *Ho*, *uHe* and *F* were conducted in GenAlEx v.6.503 (https://biology-assets.anu.edu.au/GenAlEx/Welcome.html, accessed 4 December 2016; [91]), and of *d* in MEGA v.7.0.21 (https://www.megasoftware.net/, accessed 15 March 2017; [92]).

To reconstruct the genetic structure of the analyzed group of populations, and thereby to assess the robustness of our SNPs set in terms of discrimination power for studied populations and species and to delineate more subtle genetic relationships among them, we employed a number of clustering methods. In these tests, we checked the distribution of genetic variation among different levels of the population hierarchy, i.e., among (1) the three species (herein MUS), (2) two mountain pines vs. Scots pine (MU vs. S), (3) two focal species (M vs. U), (4) dwarf vs. Scots pine (M vs. S), (5) Pyrenean vs. Scots pine (U vs. S), and (6) populations within each taxon separately afterwards (M, U, S). To investigate whether populations tend to group by species identities or other patterns, the unweighted pair group method with arithmetic mean (UPGMA) phylogenetic tree was calculated based on the mean between population distances (*d_xy_*) in MEGA, both for three species and for the two mountain pines only. Differentiation of populations was investigated by the hierarchical analysis of molecular variance (AMOVA) using Arlequin v.3.5.2.2 (http://cmpg.unibe.ch/software/arlequin35/, accessed on 15 March 2017 with updates; [93], and *Fst* statistics between pairs of populations or pairs of species were estimated in this package. We also verified these relationships by conducting two variants of multivariate analysis [94] in the adegenet package for R v.2.1.3 (http://adegenet.r-forge.r-project.org/, accessed on 13 October 2020; [95,96]). First, principal component analysis (PCA) was employed, using Edward’s Euclidean distances between samples. Next, the comparisons were repeated in principal coordinate analysis (PCoA) with analogous information on the population level, which additionally showed the distribution of genetic diversity within stands. Allele frequencies were scaled and centered prior multivariate calculations, and missing data were replaced by the mean frequencies. Additionally, to verify the hypothesis of isolation by distance (IBD) and determine if nearby populations have greater genetic similarity than those geographically separated, we employed Mantel tests [97] to determine the correlation between genetic and geographic distances among populations. The analysis was performed in adegenet, on Edward’s distances with 1000 permutations, and packages MASS v.7.3-53 (https://CRAN.R-project.org/package=MASS, accessed on 12 October 2020; [98]) and geosphere v.1.5-10 (https://cran.r-project.org/web/packages/geosphere/index.html, accessed on 12 October 2020; [99]) were used to manipulate geographical data. Finally, grouping of the samples was investigated by STRUCTURE analysis with the individual-based Bayesian clustering method implemented in [100,101,102]. In these trials, we tested all 14 populations with *K* values from 1 to 10. The admixture model with correlated allele frequencies was chosen, and no prior regarding population origin was used. For each *K*, four runs were performed, each staring with 10,000 burn-ins followed by 10,000 iterations. To determine the best *K* value and the grouping that optimally fit our data, two attempts were applied: the L(*K*) method [100] and the Evanno method based on an ad hoc measure of the rate of change in the log probability of data between subsequent *K* values—Delta*K* [103]. Both were run with STRUCTURE HARVESTER v.0.6.94 (http://taylor0.biology.ucla.edu/structureHarvester/, accessed 26 June 2020; [104]), while the results of the analyses were plotted using STRUCTURE PLOT v.2.0 (http://omicsspeaks.com/strplot2/, accessed 11 July 2020; [105]). An analogous model-based Bayesian test was performed for representatives of the *Pinus mugo* complex only to more specifically investigate the discrimination power of the array within this aggregate. In this case, *K* values between 1 and 9 were inspected, with each employing four runs with the same parameters as above.

### 4.4. Outlier SNP Detection and Functional Annotations

To identify putative selection targets differentiating inspected taxa, we searched for outlier SNPs among them, comparing allele frequencies in four setups: (1) MU vs. S, (2) M vs. S, (3) U vs. S, and (4) M vs. U. For the analysis, we used three methodological approaches, all based on *Fst* statistics, namely, FDist, BayeScan, and FLK (for comparison of the methods please see [106]). Tests implementing the FDist method were run in Arlequin; for comparisons, we set a hierarchical island model with populations nested in species. Parameters were set to 100,000 coalescent simulations, with 100 dems simulated per group and 10 groups; pairwise differences were used as the distance method for AMOVA calculations. The SNP data were considered multilocus with an unknown gametic phase, and the allowed missing level per site was changed to 0.1. For BayeScan calculations run within BayeScan v.2.1 (http://cmpg.unibe.ch/software/BayeScan/download.html, accessed 2 March 2020; [107]), and in the case of analysis implementing the FLK method in hapFLK v.1.4 (https://forge-dga.jouy.inra.fr/projects/hapflk, accessed 9 March 2020; [108]), we employed default settings. In the tests, we further focused only on the markers that differentiated our groups, concentrating on potential directional selection targets. We searched for outliers looking at two levels of significance, where *p* or *q* values, depending on the test, were set to the thresholds of 0.05 or 0.01. We were most interested in these SNPs that were detected by more than one method at the level of *p*/*q* value 0.05 and/or were confirmed on the more stringent level.

Respective contigs containing outlier SNPs revealed in those comparisons were imported into OmicsBox v.1.2 (https://www.biobam.com/omicsbox/, accessed on 11 June 2020; [109]) and analyzed for Gene Ontology (GO) terms [110,111]. Although the transcripts were already annotated in 2013 at the phase of the original RNA-Seq assembly [26], we decided to repeat the analysis for this small subset of sequences, as the GO vocabularies are continuously updated. We followed the standard procedure of (1) BLAST—finding sequences similar to the queries; in our case, blastx-fast was used, (2) GO mapping—retrieving GO terms associated with the hits obtained by BLAST, (3) gene annotation—assigning selected terms from the obtained GO pool to the query sequences, and (4) InterProScan annotation—improving the annotation results by collecting domain/motif information for analyzed query sequences. Default settings were applied, except that we narrowed BLAST searches to Viridiplantae (taxon ID: 33090). As successfully annotated, we regarded only these sequences that were blasted with high confidence (besides default filtering, we applied an additional cut-off for mean BLAST similarity above 50%), and passed two following steps, regardless of the InterPro search support. However, if InterProScan annotation was effective, the outcomes were merged with those obtained through basic gene annotation.

## Figures and Tables

**Table 1:** Genetic variation estimates of 14 populations and three pine species investigated.

Group	N	*%P*	*S_p_*	*d*	*H_o_*	*uH_e_*	*F*
M8	7	75.4	1	1262.7	0.338	0.278	−0.272
M12	22	90.2	2	1325.5	0.313	0.274	−0.123
M14	20	82.5	1	1193.4	0.292	0.253	−0.141
M16	30	91.3	4	1282.4	0.285	0.261	−0.060
U17	23	85.2	0	1368.4	0.287	0.259	−0.105
U18	42	87.5	0	1437.1	0.277	0.256	−0.075
U23	47	84.9	0	1393.2	0.245	0.247	−0.006
U24	33	85.9	1	1264.1	0.298	0.257	−0.137
U28	37	88.8	1	1236.1	0.316	0.264	−0.158
S30	22	61.1	0	906.8	0.182	0.168	−0.085
S31	37	70.6	2	946.0	0.192	0.177	−0.072
S37	44	62.5	1	867.4	0.160	0.156	−0.035
S39	32	80.3	1	994.8	0.182	0.180	0.037
S43	42	79.6	0	994.5	0.176	0.177	0.058
*P. mugo*	79	97.5	75	1308.7	0.299	0.272	−0.065
*P. uncinata*	182	96.8	12	1426.8	0.280	0.270	−0.030
*P. sylvestris*	177	92.8	25	1003.5	0.177	0.182	0.067
Total	438	100	na	1459.6	0.239	0.274	0.081

N—number of samples successfully genotyped in a group; *%P*—proportion of polymorphic sites; *Sp*—number of private polymorphisms; *d*—mean pairwise distance within group; *Ho*—observed heterozygosity; *uHe*—unbiased expected heterozygosity; *F*—fixation index; na—not applied. Population acronyms as in the Supplementary Table S1.

**Table 2:** Genetic distance and differentiation between the three investigated pine species.

Comparison	*d_xy_*	*Fst* (a/b)	Outlier SNPs (2**/2*/3*)
*P. mugo* vs. *P. uncinata*	1523.5	0.1061/0.1510	12/75/9
*P. mugo* vs. *P. sylvestris*	1761.9	0.3612/0.3937	0/1/0
*P. uncinata* vs. *P. sylvestris*	1552.3	0.2162/0.2629	12/29/6
mountain pines vs. *P. sylvestris*	1615.7	0.2324/0.2969	7/35/2

*d_xy_*—mean distance between populations from two species; *Fst*—differentiation index (calculation method: a—species considered as one group/b—hierarchical structure with populations nested in species), all results were significant at *p* value ≤ 0.001; numbers of outlier SNPs identified at: 2**—statistical significance level of *p*/*q* value ≤ 0.01 and minimum two methods simultaneously; 2*—*p*/*q* value ≤ 0.05 and minimum two methods in concert; 3*—minimum three methods at *p*/*q* value ≤ 0.05.

**Table 3:** Description of sequences containing the eight most credible outlier SNPs differentiating the two studied mountain pine species.

Sequence Name	Sequence Description and Definitions of Related GO Terms (Order: BP/MF/CC)
comp20176_c0_seq1	AP-4 complex subunit mu: protein targeting; Golgi to lysosome transport/*nd*/cytosol; cytoplasmic vesicle; trans-Golgi network; AP-4 adaptor complex; clathrin adaptor complex
comp39941_c0_seq1	glucan endo-1,3-beta-glucosidase 8-like: carbohydrate metabolic process/hydrolase activity, hydrolyzing O-glycosyl compounds/anchored component of plasma membrane
comp41821_c0_seq1	heavy metal-associated isoprenylated plant protein 36-like: metal ion transport/metal ion binding/nd
comp44835_c0_seq1	protein strictosidine synthase-like 3 biosynthetic process/strictosidine synthase activity/nd
comp50552_c0_seq1	phosphatidate phosphatase PAH2-like: hydrolase activity/cellular metabolic process/nd
comp52994_c0_seq1	unknown: transcription coregulator activity/regulation of transcription, DNA-templated/nd
comp53610_c0_seq1	aminodeoxychorismate synthase, chloroplastic isoform X1: biosynthetic process; carboxylic acid metabolic process/nd/nd
comp54487_c0_seq1	serine/threonine-protein kinase VPS15-like isoform X1: protein kinase activity; protein binding; endosome; autophagy/vacuolar transport; phosphorylation/phosphatidylinositol 3-kinase complex, class III; intracellular transport

BP—Biological Process; CC—Cellular Component; MF—Molecular Function; nd—no data for particular GO domain.

## Data Availability

The dataset supporting the results of this publication is available from the corresponding author upon request.

## Supplementary Materials

The following are available online at https://www.mdpi.com/article/10.3390/ijms22073477/s1. Table S1: List and description of 14 source populations from which offspring genotypes were investigated in the study; Figure S1: Shared and unique SNP polymorphisms segregating in populations of dwarf, Pyrenean and Scots pines; Table S2: Distances and differentiation between investigated pine populations measured by (a) *d_xy_*—mean number of differences between groups, (b) *Fst*—inbreeding coefficient within subpopulations; Figure S2: Evolutionary relationships of populations inferred using the UPGMA method calculated on the mean between group distances (*d_xy_*) for (a) three studied taxa, (b) two mountain pines only; Figure S3: Principal coordinate analysis (PCoA) revealing relationships between 14 studied populations. (a) Discrimination among three pine species, (b) distinction within the *P. mugo* complex; Figure S4: Results of clustering analysis performed with STRUCTURE for 14 studied populations. (a) Delta*K* estimates and (b) mean values of natural logarithm of likelihood, as indicated by STRUCTURE HARVESTER for *K* groups between 1 and 10. Detailed results of analysis for specific *K* values: (c) *K* = 6, (d) *K* = 7, (e) *K* = 8, (f) *K* = 9, (g) *K* = 10; Figure S5: Results of clustering analysis performed with STRUCTURE for 9 populations of *P. mugo* complex. (A) Delta*K* estimates and (b) mean values of the natural logarithm of likelihood, as indicated by STRUCTURE HARVESTER for *K* groups between 1 and 9. Detailed results of analysis for specific *K* values: (c) *K* = 4, (d) *K* = 5, (e) *K* = 6, (f) *K* = 7, (g) *K* = 8 and (h) *K* = 9; Table S3: Results of Mantel tests verifying the isolation by distance (IBD) hypothesis performed on Edwards’ genetic and log standardized geographic distances for different combinations of studied pine populations; Figure S6: Correlation plots of the geographic and genetic distances verifying the isolation by distance (IBD) hypothesis in three pine species. (a) Mantel test including comparisons within and between populations of all species, (b) results of three independent tests performed within taxa; Table S4: Gene ontology (GO) annotation of sequences with outlier polymorphisms detected; Table S5: Frequencies of outlier SNP alleles; Figure S7: Distribution of the most common third-level gene ontologies annotated for 64 sequences in which outlier SNPs were detected between dwarf and Pyrenean pine, shown by domains.
