# Supplementary material for: Candidate Genes for the High-Altitude Adaptations of Two Mountain Pine Taxa
Source: Int J Mol Sci. 2021 Mar 27;22(7):3477. doi: 10.3390/ijms22073477 (PMC8036860; doi:10.3390/ijms22073477)
Supplement: Supplementary file 1 [file ijms-22-03477-s001.pdf]

Table S1. List and descriptions of the 14 source populations from which offspring genotypes were investigated in the study.

Tax.	Acr.	Geographic localization (country code)	Latitude	Longitude	Alt.	N
P. mugo	M8	Dinaric Alps, Durmitor Mts., Meded (ME)	43°09'33" N	19°05'27" E	2100	23
	M12	Northern Limestone Alps, Karwendel Mts., Scharnitz (AT)	47°22'42" N	11°17'45" E	1400	42
	M14	Carnic Alps, Nassfeld Pass, Pontebba (IT)	46°32'45" N	13°15'35" E	1530	37
	M16	Central Apennines, Majella massif, Barrea (IT)	41°46'20" N	13°58'30" E	2200	39
P. uncinata	U17	Eastern Pyrenees, Vall de Ransol (AD)	42°35'02" N	01°38'21" E	2025	26
	U18	Eastern Pyrenees, Engolasters (AD)	42°31'28" N	01°34'12" E	2000	42
	U23	Western Pyrenees, La Trapa near Jaca (ES)	42°41'19" N	00°32'12" W	1720	47
	U24	Sierra de Gudar, Valdelinares (ES)	40°28'49" N	00°41'51" W	2000	40
	U28	Massif Central, Col de la Croix-Morand (FR)	45°36'00" N	02°50'59" E	1400	46
P. sylvestris	S30	Scottish Highlands, Wester Ross, Shieldaig (GB)	57°30'35" N	05°38'24" W	81	22
	S31	Eastern Scottland, Glen Tanar (GB)	57°02'60" N	02°51'36" W	160	38
	S37	Sierra Nevada, Trevenque (ES)	37°05'47" N	03°32'51" W	1170	45
	S39	Finnish Lakeland, Punkaharju (FI)	61°45'33" N	29°23'21" E	80	34
	S43	Polish Plains, Jarocin (PL)	51°58'20" N	17°28'40" E	120	43

Tax. – taxon name; Acr. – acronym for the population; Alt. - altitude given in meters above sea level; N – number of sampled specimens.

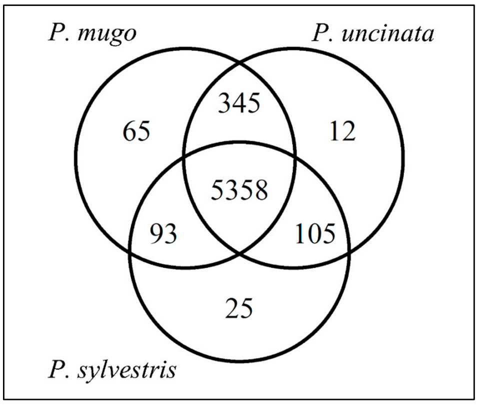

Figure S1. Shared and unique SNP polymorphisms segregating in populations of dwarf, Pyrenean and Scots pines.

Table S2. Distances and differentiation between investigated pine populations measured by (**a**) d_{xy} - mean number of differences between groups, (**b**) *Fst* - inbreeding coefficient within subpopulations.

(**a**)

	**M8**	**M12**	**M14**	**M16**	**U17**	**U18**	**U23**	**U24**	**U28**	**S30**	**S31**	**S37**	**S39**
**M12**	1313.3												
**M14**	1272.2	1311.5											
**M16**	1307.6	1357.8	1312.1										
**U17**	1454.1	1481.2	1484.0	1488.3									
**U18**	1495.4	1522.7	1524.7	1528.5	1443.5								
**U23**	1525.1	1554.7	1565.6	1562.5	1465.8	1512.1							
**U24**	1474.9	1518.3	1541.5	1536.5	1440.7	1488.6	1459.1						
**U28**	1442.2	1491.1	1519.7	1514.1	1418.4	1468.1	1438.0	1270.9					
**S30**	1681.9	1730.0	1797.8	1775.1	1635.3	1697.4	1610.4	1436.1	1343.7				
**S31**	1671.9	1722.1	1788.4	1763.0	1628.5	1694.8	1607.6	1432.8	1338.3	972.1			
**S37**	1697.8	1745.7	1817.5	1790.5	1637.3	1695.9	1605.8	1436.3	1342.6	1006.4	1015.4		
**S39**	1672.8	1723.6	1788.7	1765.8	1642.0	1702.7	1624.2	1451.0	1358.6	1017.1	1013.6	1055.1	
**S43**	1687.1	1737.7	1803.3	1779.9	1649.1	1710.5	1626.3	1453.2	1360.1	1009.8	1010.4	1041.6	1023.1

(**b**)

	**M8**	**M12**	**M14**	**M16**	**U17**	**U18**	**U23**	**U24**	**U28**	**S30**	**S31**	**S37**	**S39**
**M12**	0.015												
**M14**	0.039	0.042											
**M16**	0.029	0.042	0.058										
**U17**	0.096	0.093	0.140	0.114									
**U18**	0.093	0.093	0.136	0.113	0.028								
**U23**	0.127	0.127	0.173	0.147	0.060	0.065							
**U24**	0.149	0.153	0.208	0.177	0.089	0.093	0.090						
**U28**	0.141	0.147	0.205	0.174	0.085	0.089	0.085	0.017					
**S30**	0.382	0.358	0.422	0.381	0.305	0.295	0.270	0.240	0.196				
**S31**	0.370	0.354	0.415	0.377	0.302	0.295	0.269	0.233	0.186	0.048			
**S37**	0.413	0.391	0.451	0.412	0.335	0.322	0.294	0.263	0.218	0.121	0.109		
**S39**	0.348	0.335	0.396	0.359	0.287	0.280	0.258	0.222	0.178	0.066	0.044	0.119	
**S43**	0.355	0.344	0.403	0.368	0.294	0.288	0.263	0.226	0.180	0.058	0.041	0.107	0.029

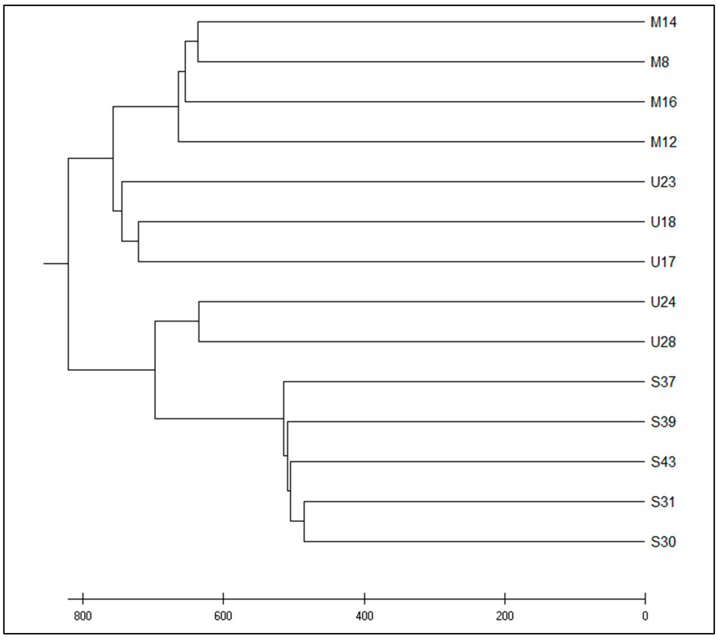

(**a**)

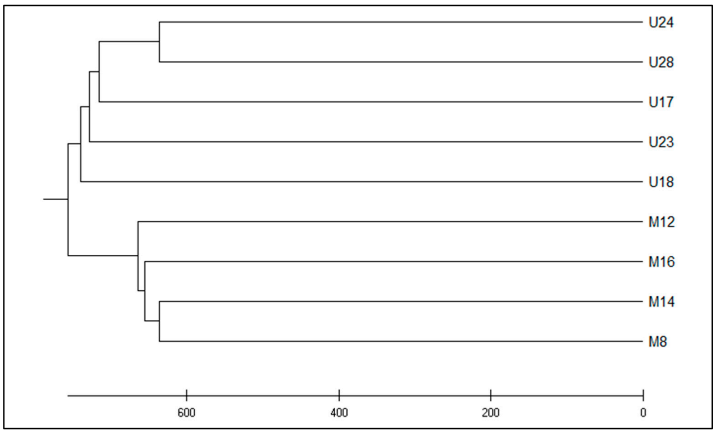

(**b**)

Figure S2. Evolutionary relationships of populations inferred using the UPGMA method calculated on the mean between group distances (d_{xy}) for (**a**) three studied taxa, (**b**) two mountain pines only.

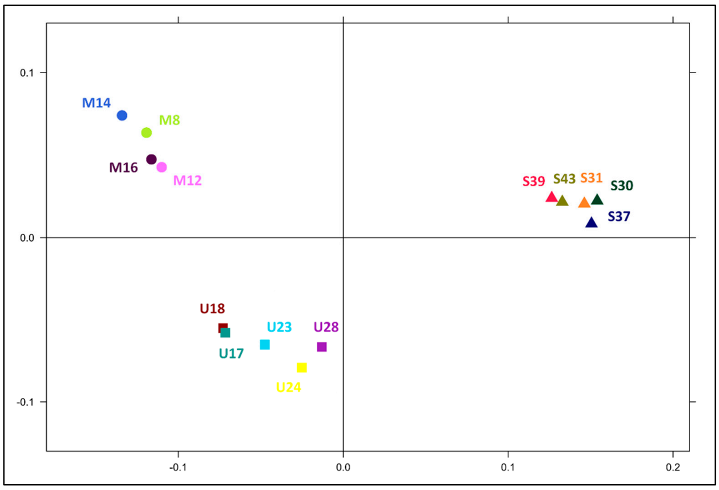

(**a**)

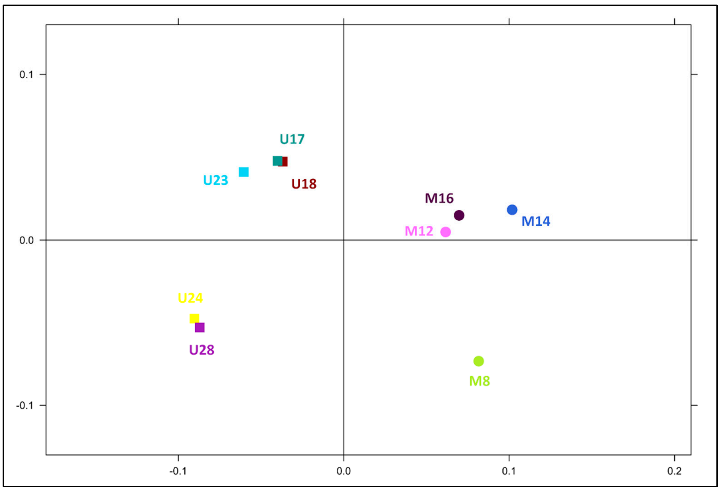

(**b**)

Figure S3. Principal coordinate analysis (PCoA) revealing the relationships between the 14 studied populations. (**a**) Discrimination among three pine species, horizontal axis corresponds to 58.8% of variation and vertical axis represents further 12.6%; (**b**) distinction within the *P. mugo* complex with 44.5% of variation distributed along horizontal and 16.0% along vertical axis.

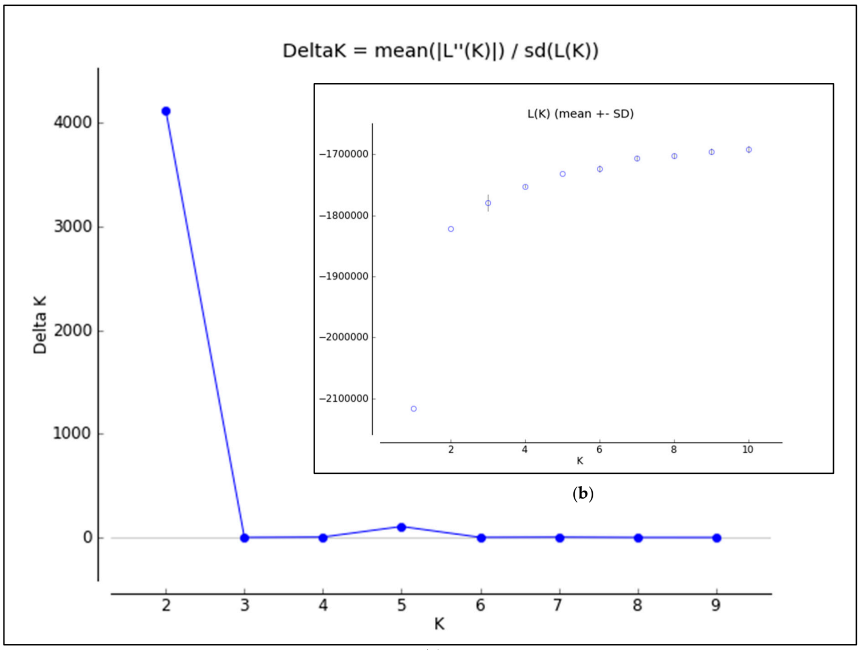
DeltaK = mean(|L''(K)|) / sd(L(K))
Delta K
4000
3000
2000
1000
0
2 3 4 5 6 7 8 9
K
L(K) (mean +- SD)
−1700000
−1800000
−1900000
−2000000
−2100000
2 4 6 8 10
K

(b)

(a)

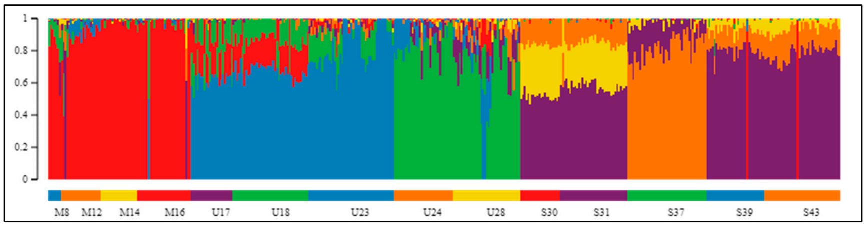
1
0.8
0.6
0.4
0.2
0
M8 M12 M14 M16 U17 U18 U23 U24 U28 S30 S31 S37 S39 S43

(c)

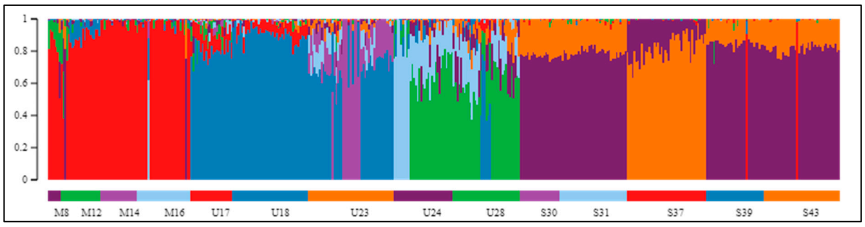
1
0.8
0.6
0.4
0.2
0
M8 M12 M14 M16 U17 U18 U23 U24 U28 S30 S31 S37 S39 S43

(d)

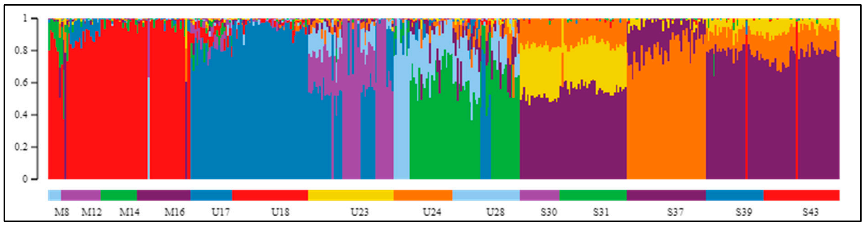
1
0.8
0.6
0.4
0.2
0
M8 M12 M14 M16 U17 U18 U23 U24 U28 S30 S31 S37 S39 S43

(e)

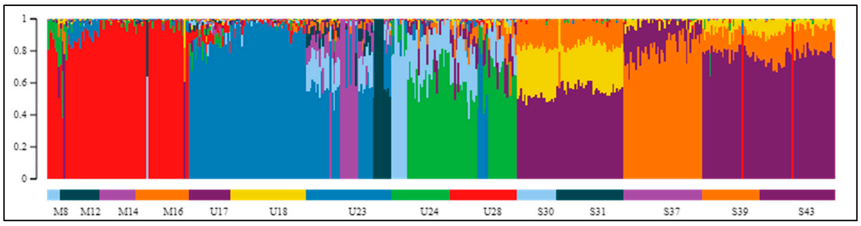

(**f**)

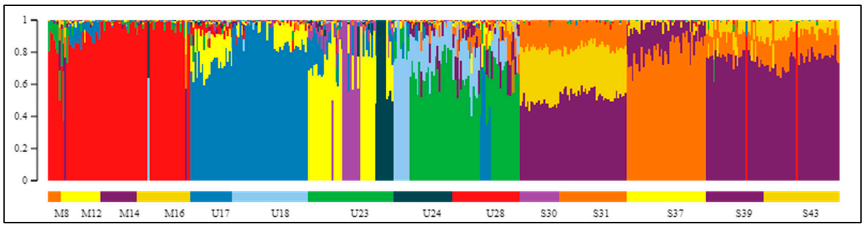

(**g**)

Figure S4. Results of clustering analysis performed with STRUCTURE for the 14 studied populations. (**a**) Delta*K* estimates and (**b**) mean values of natural logarithm of likelihood, as indicated by STRUCTURE HARVESTER for *K* groups between 1 and 10. Detailed results of analysis for specific *K* values: (**c**) *K* = 6, (**d**) *K* = 7, (**e**) *K* = 8, (**f**) *K* = 9, (**g**) *K* = 10. Scale on the left and vertical bars represent proportion of each genome being composed by variants specific for particular genomes. Horizontal colour bars at the bottom of each chart label individuals from distinct populations.

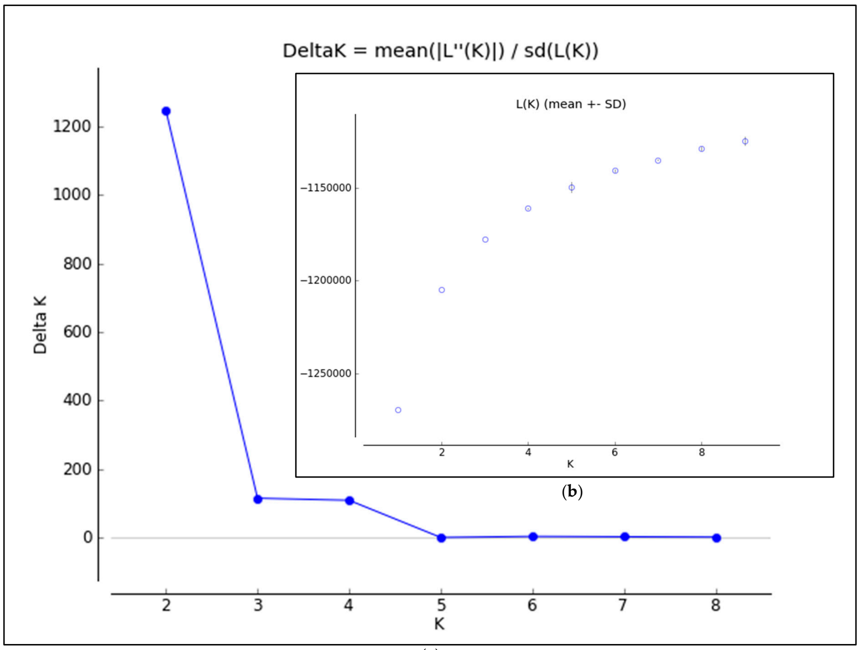
DeltaK = mean(|L''(K)|) / sd(L(K))
Delta K
K
L(K) (mean +- SD)
K

(b)

(a)

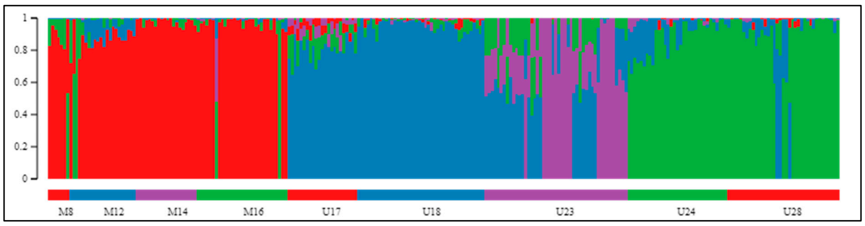
M8
M12
M14
M16
U17
U18
U23
U24
U28

(c)

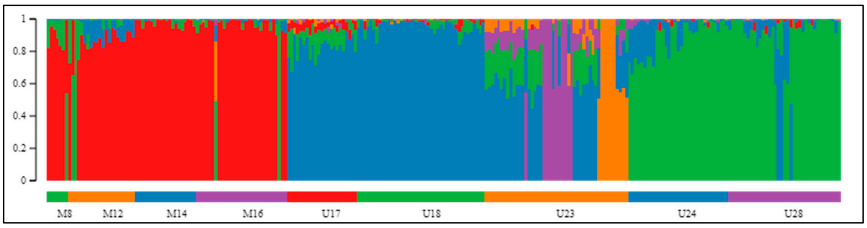
M8
M12
M14
M16
U17
U18
U23
U24
U28

(d)

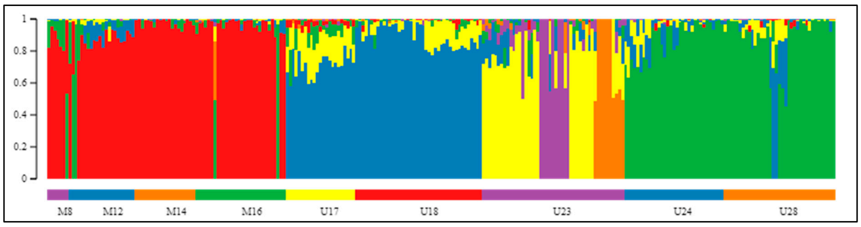
M8
M12
M14
M16
U17
U18
U23
U24
U28

(e)

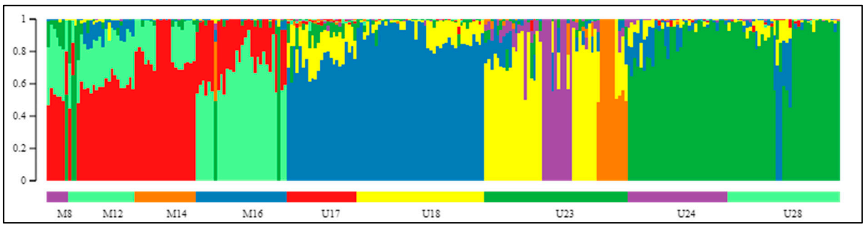

(**f**)

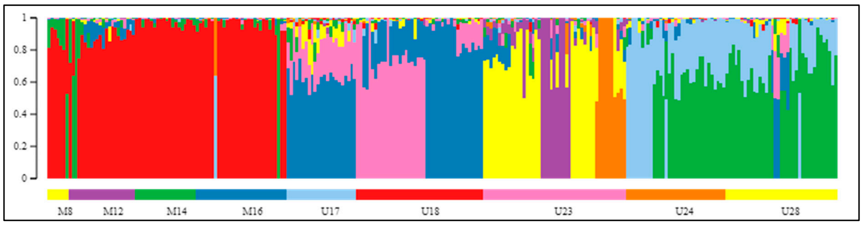

(**g**)

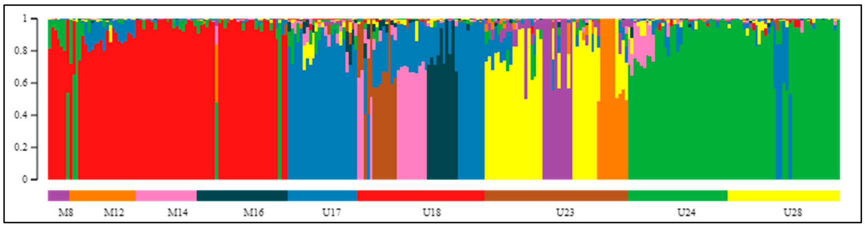

(**h**)

Figure S5. Results of clustering analysis performed with STRUCTURE for 9 populations of *Pinus mugo* complex. (**a**) Delta*K* estimates and (**b**) mean values of the natural logarithm of likelihood, as indicated by STRUCTURE HARVESTER for *K* groups between 1 and 9. Detailed results of analysis for specific *K* values: (**c**) *K* = 4, (**d**) *K* = 5, (**e**) *K* = 6, (**f**) *K* = 7, (**g**) *K* = 8 and (**h**) *K* = 9. Scale on the left and vertical bars represent proportion of each genome being composed by variants specific for particular genomes. Horizontal colour bars at the bottom of each chart label individuals from distinct populations.

Table S3. Results of Mantel tests verifying the isolation by distance (IBD) hypothesis performed on Edwards' genetic and log standardized geographic distances for different combinations of studied pine populations.

**Populations analysed**	**N**	**r**	***p* value**
Three species	14	0.465	0.001
P. mugo & *P. uncinata*	9	0.705	0.005
P. mugo & *P. sylvestris*	9	0.335	0.021
P. uncinata & *P. sylvestris*	10	0.398	0.014
within *P. mugo*	4	0.277	0.291
within *P. uncinata*	5	0.382	0.142
within *P. sylvestris*	5	0.506	0.109

N – number of populations tested, r - Pearson correlation coefficient.

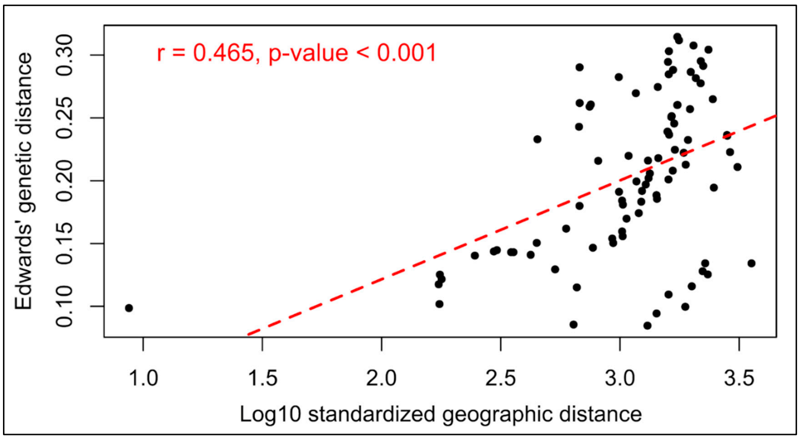

(**a**)

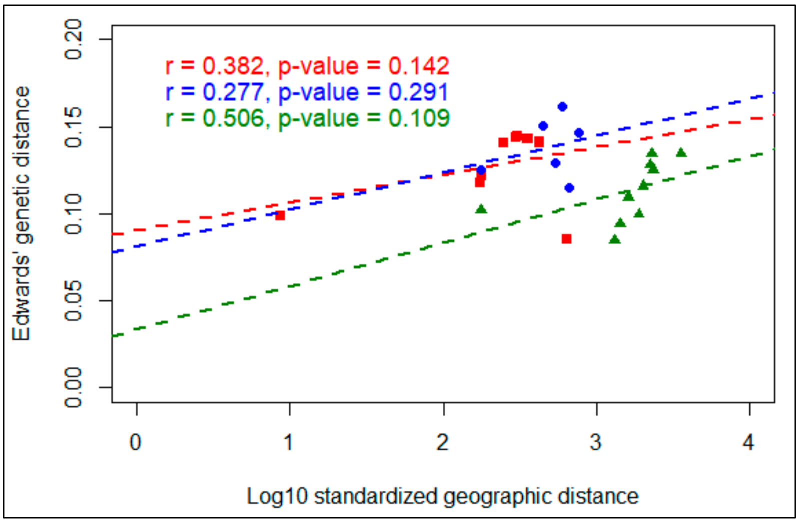

(**b**)

Figure S6. Correlation plots of the geographic and genetic distances verifying the isolation by distance (IBD) hypothesis in three pine species. (**a**) Mantel test including comparisons within and between populations of all species, (**b**) results of three independent tests performed within taxa: blue dots – dwarf pine, red squares – Pyrenean pine, green triangles – Scots pine. Regression lines and results of the tests on bottom panel are indicated with respective colours.

Table S4. Gene ontology (GO) annotation of sequences with outlier polymorphisms detected. The green background distinguishes the sequence differentiating Scots pine from two mountain pines while markers differentiating the latter are fulfilled with blue; eight sequences with the most reliable outlier SNPs supported by all three methods and significant at *p*/*q* value < 0.01 are given in bold.

Sequence name	Species comparison				Gene Ontology (GO) annotation	
(outlier SNP position)	M *vs* U	MU *vs* S	M *vs* S	U *vs* S	Domains	IDs
comp17013_c0_seq1 (1608)	X*				BP; CC	GO:0005774;GO:0016021;GO:0055085
comp18757_c0_seq1 (2058)		X[3]*		X[3]	BP; MF	GO:0008760;GO:0019277
comp18889_c0_seq1 (111)	X				BP; MF	GO:0016567;GO:0016874;GO:0061630
comp18988_c0_seq1 (2220)		X*		X*	BP; CC; MF	GO:0000287;GO:0004497;GO:0005524;GO:0009535;GO:0009853;GO:0015986; GO:0016984;GO:0019253;GO:0045261;GO:0046933;GO:0055114
comp19056_c0_seq1 (1447)	X				BP; MF	GO:0003712;GO:0006355
comp19359_c0_seq1 (402)				X	BP; CC; MF	GO:0005506;GO:0009055;GO:0009507;GO:0016021;GO:0022900;GO:0043448
comp19450_c0_seq1 (1154)		X		X	BP; MF	GO:0004252;GO:0006508
comp19472_c0_seq1 (739)	X				CC	GO:0016021
comp19700_c0_seq1 (1179)	X				BP; CC; MF	GO:0003677;GO:0003700;GO:0005634;GO:0006355
comp20014_c0_seq1 (2275)	X				BP; CC; MF	GO:0004675;GO:0005515;GO:0005524;GO:0005886;GO:0005975;GO:0006468
comp20164_c0_seq1 (232)	X				BP; MF	GO:0004857;GO:0043086
**comp20176_c0_seq1 (240)**	**X[3]***				**BP; CC**	**GO:0005802;GO:0005829;GO:0006605;GO:0030124;GO:0030131;GO:0031410; GO:0090160**
comp20234_c0_seq1 (1126)	X				BP; CC	GO:0005739;GO:0016554;GO:0080156
comp24802_c0_seq1 (1869)	X				BP; MF	GO:0005985;GO:0016157;GO:0046524
comp25462_c0_seq1 (100)	X				BP; CC; MF	GO:0001671;GO:0005829;GO:0006457;GO:0032781;GO:0051087;GO:0051879
comp261721_c0_seq1 (255)	X				CC	GO:0016020
comp27292_c0_seq1 (1521)		X			BP; CC; MF	GO:0002184;GO:0005829;GO:0016149;GO:0018444;GO:1990825
comp28590_c0_seq1 (1341)		X*	X	X*	BP; CC; MF	GO:0000287;GO:0008137;GO:0009055;GO:0009522;GO:0009535;GO:0015979; GO:0016021;GO:0016168;GO:0018298;GO:0022900;GO:0048038;GO:0051539
comp29118_c0_seq1 (688)	X				BP; CC; MF	GO:0006629;GO:0016021;GO:0016491;GO:0055114
comp29127_c0_seq1 (671)		X			BP; CC; MF	GO:0000209;GO:0005515;GO:0005524;GO:0005783;GO:0016021;GO:0016874; GO:0030433;GO:0042631;GO:0061631;GO:1902457
comp30050_c0_seq1 (910)		X		X	BP; MF	GO:0003700;GO:0006352;GO:0006355
comp31542_c0_seq1 (466)	X				BP; CC; MF	GO:0003700;GO:0005634;GO:0006355;GO:0009739;GO:0043565
comp31770_c0_seq1 (1348)	X				BP; MF	GO:0003700;GO:0006355
comp33894_c0_seq1 (1936)				X	BP; CC; MF	GO:0004672;GO:0005515;GO:0005524;GO:0006468;GO:0016020;GO:0016491
comp34171_c0_seq1 (187)	X				BP; CC; MF	GO:0004674;GO:0005524;GO:0005634;GO:0006357;GO:0006468
comp35189_c0_seq1 (1282)	X				BP; CC; MF	GO:0003333;GO:0005886;GO:0015171;GO:0016021
comp35641_c0_seq1 (1756)	X				MF	GO:0046983

comp35702_c0_seq1 (1216)		X		BP; MF	GO:0004190;GO:0006508;GO:0006629;GO:0030163
comp358563_c0_seq1 (135)		X		BP; CC	GO:0010005;GO:0043622
comp36656_c0_seq1 (1022)	X			CC	GO:0009507
comp36690_c1_seq1 (361)	X			CC; MF	GO:0005794;GO:0008168;GO:0016020
comp36863_c0_seq1 (1165)		X	X*	BP; CC; MF	GO:0005515;GO:0005643;GO:0005654;GO:0005829;GO:0006607;GO:0008139; GO:0042025;GO:0061608
comp37364_c0_seq1 (613)	X			CC	GO:0016021
comp37406_c0_seq1 (254)		X		BP; CC	GO:0043622;GO:0110165
comp37651_c0_seq1 (1062)		X*	X*	BP; MF	GO:0016206;GO:0019438;GO:0032259;GO:0046983;GO:0102084;GO:0102938
comp38584_c0_seq1 (1237)		X^{3}*	X	BP; CC; MF	GO:0004674;GO:0005524;GO:0005634;GO:0005737;GO:0005886;GO:0006468
comp38629_c0_seq1 (349)	X			BP; CC; MF	GO:0005506;GO:0016021;GO:0016705;GO:0020037;GO:0055114
comp38943_c0_seq1 (866)		X^{3}*	X^{3}*	BP; CC; MF	GO:0005634;GO:0043565;GO:0045893;GO:0046982
**comp39941_c0_seq1 (1816)**	**X^{3}***			**BP; CC; MF**	**GO:0004553;GO:0005975;GO:0046658**
comp40569_c0_seq1 (764)			X	BP; CC; MF	GO:0004656;GO:0005506;GO:0005783;GO:0016021;GO:0018401;GO:0031418; GO:0055114
comp41803_c0_seq1 (1452)	X			BP; CC; MF	GO:0005737;GO:0006511;GO:0016021;GO:0043231;GO:0061630
**comp41821_c0_seq1 (413)**	**X^{3}***			**BP; MF**	**GO:0030001;GO:0046872**
comp423545_c0_seq1 (183)	X			BP; MF	GO:0004190;GO:0006508
comp42606_c0_seq1 (1061)		X^{3}*	X^{3}*	BP; MF	GO:0009055;GO:0015035;GO:0022900;GO:0045454
comp42726_c0_seq1 (307)		X^{3}*	X^{3}*	BP; MF	GO:0004252;GO:0006508
comp42805_c1_seq1 (2699)	X			BP; MF	GO:0003700;GO:0006355;GO:0043565
comp42950_c0_seq1 (425)	X*			BP; MF	GO:0000155;GO:0000160;GO:0006355;GO:0009584;GO:0009585;GO:0009881; GO:0017006;GO:0018298;GO:0023014;GO:0042803
comp43820_c0_seq1 (103)		X*	X*	BP; MF	GO:0006482;GO:0016740;GO:0051723
comp44465_c0_seq1 (1110)	X			BP; MF	GO:0016702;GO:0046872;GO:0055114
**comp44835_c0_seq1 (339)**	**X^{3}***			**BP; MF**	**GO:0009058;GO:0016844**
comp44981_c0_seq1 (281)	X			CC	GO:0016021
comp45024_c0_seq1 (839)		X		MF	GO:0003677
comp45463_c0_seq1 (2507)		X		BP; MF	GO:0008143;GO:0043488;GO:1900364
comp45510_c0_seq1 (218)	X			MF	GO:0003824
comp45924_c0_seq1 (434)			X	BP; MF	GO:0003712;GO:0009910;GO:1903506
comp46344_c0_seq1 (1108)		X		BP; CC	GO:0016020;GO:0071782;GO:0071786
comp47007_c0_seq1 (1300)	X			BP	GO:0001522
comp47328_c0_seq1 (518)	X			BP; CC; MF	GO:0006857;GO:0015333;GO:0016021;GO:0042937;GO:0055085
comp47467_c0_seq1 (1161)		X		BP; CC; MF	GO:0005525;GO:0005737;GO:0006886;GO:0016192
comp47663_c0_seq1 (1253)			X	CC; MF	GO:0005737;GO:0008168;GO:0016020

comp48051_c0_seq1 (220)	X			CC	GO:0016020
comp48330_c0_seq1 (140)		X[3]*	X[3]*	BP; MF	GO:0004096;GO:0005515;GO:0020037;GO:0055114;GO:0098869
comp48723_c0_seq2 (83)	X			BP; CC; MF	GO:0003735;GO:0006364;GO:0006412;GO:0022627;GO:0032040;GO:0042274
comp48942_c0_seq1 (1943)	X			BP; MF	GO:0003700;GO:0006355;GO:0043565
comp49679_c0_seq1 (4572)	X			BP; MF	GO:0004674;GO:0005524;GO:0018105;GO:0018107
comp49745_c0_seq1 (703)	X			BP	GO:0006355
comp49772_c0_seq1 (1003)	X[3]			BP; MF	GO:0006807;GO:0016151
comp50296_c0_seq1 (1883)	X			BP; CC; MF	GO:0001193;GO:0003676;GO:0003899;GO:0005665;GO:0005730;GO:0006283; GO:0006367;GO:0006379;GO:0008270;GO:0016021
comp50473_c0_seq1 (669)	X			BP; CC; MF	GO:0003700;GO:0005634;GO:0030154;GO:0043565
**comp50552_c0_seq1 (1098)**	**X[3]***			**BP; MF**	**GO:0016787;GO:0044237**
comp50851_c0_seq1 (996)		X		MF	GO:0000166;GO:0047710
comp50905_c0_seq4 (942)			X	BP; MF	GO:0006479;GO:0008276
comp51215_c0_seq1 (1350)	X			BP; MF	GO:0006810;GO:0022857
comp51336_c0_seq1 (1958)	X			BP; CC; MF	GO:0004497;GO:0005506;GO:0016021;GO:0016705;GO:0020037;GO:0055114
comp51669_c0_seq1 (313)	X			BP; CC; MF	GO:0003723;GO:0005654;GO:0043484;GO:1990904
comp51783_c0_seq1 (508)	X			BP; CC; MF	GO:0005829;GO:0009854;GO:0016618;GO:0030267;GO:0047995;GO:0051287; GO:0055114;GO:0102742
comp52585_c0_seq1 (127)	X			BP; CC; MF	GO:0004160;GO:0005507;GO:0009082;GO:0009553;GO:0009555;GO:0009570; GO:0009651;GO:0048364
comp52700_c0_seq1 (1559)	X			CC; MF	GO:0003677;GO:0005634;GO:0042025;GO:0046872
**comp52994_c0_seq1 (245)**	**X[3]***			**BP; MF**	**GO:0003712;GO:0006355**
comp53206_c1_seq1 (1087)	X			CC; MF	GO:0003924;GO:0005525;GO:0005739
comp53528_c0_seq1 (1313)	X			MF	GO:0003676
comp53591_c0_seq1 (320)	X			CC; MF	GO:0003677;GO:0005515;GO:0005634;GO:0042025
**comp53610_c0_seq1 (276)**	**X[3]***			**BP**	**GO:0009058;GO:0019752**
comp53749_c0_seq7 (2413)		X*	X*	BP; MF	GO:0003676;GO:0006397;GO:0046872
comp53870_c0_seq1 (109)	X			BP; CC; MF	GO:0004222;GO:0006508;GO:0006518;GO:0009507;GO:0016021;GO:0046872
comp54118_c0_seq1 (2585)	X			MF	GO:0005515
**comp54487_c0_seq1 (3338)**	**X[3]***			**BP; CC; MF**	**GO:0004672;GO:0005515;GO:0005768;GO:0006914;GO:0007034;GO:0016310; GO:0035032;GO:0046907**
comp55083_c0_seq1 (1074)	X			MF	GO:0003676
comp56793_c0_seq1 (670)			X	BP; CC; MF	GO:0006605;GO:0009306;GO:0009535;GO:0015450;GO:0016021;GO:0071806
comp57453_c0_seq1 (2049)		X	X	BP	GO:0007275;GO:0030154
comp58612_c0_seq1 (681)	X*			BP; CC	GO:0005789;GO:0005886;GO:0016021;GO:0061817;GO:0090158
comp59746_c0_seq1 (1253)		X		BP; CC; MF	GO:0005829;GO:0006508;GO:0016920

comp59967_c0_seq1 (1910)		X³		X	BP; MF	GO:0003700;GO:0006355
comp60629_c0_seq1 (1021)	X				BP; CC; MF	GO:0015095;GO:0015693;GO:0016021
comp61109_c0_seq1 (1237)		X		X*	MF	GO:0005515
comp63308_c0_seq1 (1097)	X*				BP; CC	GO:0000398;GO:0000932;GO:0005688;GO:0046540;GO:0071011;GO:0071013; GO:1990726
comp64225_c0_seq1 (1194)	X				MF	GO:0003700
comp71632_c0_seq1 (1208)	X				BP; CC; MF	GO:0000014;GO:0000110;GO:0000413;GO:0000712;GO:0000720;GO:0000724; GO:0003684;GO:0003697;GO:0003755;GO:0006296;GO:0006979;GO:0009314; GO:0016021;GO:1901255
comp72132_c0_seq1 (584)	X				BP; CC; MF	GO:0000166;GO:0003700;GO:0005634;GO:0006355;GO:0009536;GO:0009739; GO:0016874;GO:0043565
comp74829_c0_seq1 (165)		X			BP; CC; MF	GO:0005768;GO:0005802;GO:0008757;GO:0016021;GO:0032259;GO:0052546
comp1178_c0_seq1 (146)		X		X	*undetermined*	
comp166107_c0_seq1 (81)	X				*undetermined*	
comp19558_c0_seq1 (688)		X*			*undetermined*	
comp21559_c0_seq1 (165)	X				*undetermined*	
comp227564_c0_seq1 (556)		X			*undetermined*	
comp29071_c0_seq1 (277)	X				*undetermined*	
comp37643_c0_seq1 (1161)	X				*undetermined*	
comp39687_c0_seq1 (706)		X			*undetermined*	
comp40831_c0_seq1 (622)				X	*undetermined*	
comp43127_c0_seq1 (312)				X	*undetermined*	
comp45714_c0_seq1 (1064)				X	*undetermined*	
comp48574_c0_seq1 (1639)	X				*undetermined*	
comp49514_c0_seq1 (63)		X			*undetermined*	
comp49840_c0_seq1 (2135)		X		X³*	*undetermined*	
comp51626_c0_seq1 (1245)	X				*undetermined*	
comp52601_c0_seq1 (205)	X				*undetermined*	
comp58030_c0_seq1 (550)	X				*undetermined*	
comp66672_c0_seq1 (661)	X				*undetermined*	
0_4394_01_1 (522)	X				*undetermined*	
CL12234_1_1 (626)	X				*undetermined*	

X - SNP statistically significant at *p/q* value of 0.05 in at least two tests; X³ - significant at *p/q* value of 0.05 in all three tests; X* - significant at *p/q* value of 0.01 in at least two tests; M *vs* U – dwarf pine *vs* Pyrenean pine; MU *vs* S – mountain taxa *vs* Scots pine; M *vs* S – dwarf *vs* Scots pine; U *vs* S – Pyrenean *vs* Scots pine.

Deleted: *e*

Table S5. Frequency of alleles in the loci with outlier SNPs identified. The green background distinguishes the sequence differentiating Scots pine from two mountain pines while markers differentiating the latter are fulfilled with blue; eight sequences with the most reliable outlier SNPs supported by all three methods and significant at *p*/*q* value < 0.01 are given in bold.

Sequence name (SNP)	Allele pair	*P. mugo*	*P. uncinata*	*P. mugo* complex	*P. sylvestris*
comp17013_c0_seq1 (1608)	G / A	0.02 / 0.98	0.22 / 0.78	0.15 / 0.85	0.33 / 0.67
comp18757_c0_seq1 (2058)	G / A	0.83 / 0.17	0.9 / 0.1	0.88 / 0.12	0.99 / 0.01
comp18889_c0_seq1 (111)	C / T	0.08 / 0.92	0.15 / 0.85	0.13 / 0.87	0.13 / 0.87
comp18988_c0_seq1 (2220)	C / A	0.5 / 0.5	0.75 / 0.25	0.67 / 0.33	0.72 / 0.28
comp19056_c0_seq1 (1447)	G / T	0.12 / 0.88	0.35 / 0.65	0.28 / 0.72	0 / 1
comp19359_c0_seq1 (402)	A / G	0.9 / 0.1	0.89 / 0.11	0.89 / 0.11	1 / 0
comp19450_c0_seq1 (1154)	G / A	0.87 / 0.13	0.95 / 0.05	0.93 / 0.07	1 / 0
comp19472_c0_seq1 (739)	A / T	0.28 / 0.72	0.18 / 0.82	0.21 / 0.79	0.01 / 0.99
comp19700_c0_seq1 (1179)	C / A	0.89 / 0.11	0.94 / 0.06	0.93 / 0.07	0.99 / 0.01
comp20014_c0_seq1 (2275)	C / T	0.56 / 0.44	0.36 / 0.64	0.42 / 0.58	0.43 / 0.57
comp20164_c0_seq1 (232)	C / A	0.79 / 0.21	0.98 / 0.02	0.93 / 0.07	1 / 0
**comp20176_c0_seq1 (240)**	**T / C**	**0.05 / 0.95**	**0.31 / 0.69**	**0.23 / 0.77**	**0.6 / 0.4**
comp20234_c0_seq1 (1126)	G / A	0.01 / 0.99	0.03 / 0.97	0.03 / 0.97	0.11 / 0.89
comp24802_c0_seq1 (1869)	C / G	0.17 / 0.83	0.04 / 0.96	0.08 / 0.92	0.04 / 0.96
comp25462_c0_seq1 (100)	T / G	0.82 / 0.18	0.85 / 0.15	0.84 / 0.16	0.9 / 0.1
comp261721_c0_seq1 (255)	A / G	0.01 / 0.99	0.02 / 0.98	0.02 / 0.98	0.03 / 0.97
comp27292_c0_seq1 (1521)	C / T	0.17 / 0.83	0.49 / 0.51	0.4 / 0.6	0.42 / 0.58
comp28590_c0_seq1 (1341)	C / A	0.79 / 0.21	0.87 / 0.13	0.85 / 0.15	0.78 / 0.22
comp29118_c0_seq1 (688)	C / G	0.05 / 0.95	0.03 / 0.97	0.03 / 0.97	0.01 / 0.99
comp29127_c0_seq1 (671)	A / G	0.98 / 0.02	0.95 / 0.05	0.96 / 0.04	1 / 0
comp30050_c0_seq1 (910)	T / C	0.67 / 0.33	0.58 / 0.42	0.61 / 0.39	0.83 / 0.17
comp31542_c0_seq1 (466)	G / C	0.9 / 0.1	0.94 / 0.06	0.93 / 0.07	0.75 / 0.25
comp31770_c0_seq1 (1348)	G / C	0.07 / 0.93	0.06 / 0.94	0.06 / 0.94	0.08 / 0.92
comp33894_c0_seq1 (1936)	A / G	0.93 / 0.07	0.8 / 0.2	0.83 / 0.17	0.5 / 0.5
comp34171_c0_seq1 (187)	C / T	0.32 / 0.68	0.36 / 0.64	0.34 / 0.66	0.2 / 0.8
comp35189_c0_seq1 (1282)	G / C	0.09 / 0.91	0.02 / 0.98	0.04 / 0.96	0.02 / 0.98
comp35641_c0_seq1 (1756)	G / T	0.93 / 0.07	0.96 / 0.04	0.95 / 0.05	0.93 / 0.07
comp35702_c0_seq1 (1216)	C / A	0.39 / 0.61	0.44 / 0.56	0.42 / 0.58	0.03 / 0.97
comp358563_c0_seq1 (135)	A / G	0.66 / 0.34	0.91 / 0.09	0.84 / 0.16	0.97 / 0.03
comp36656_c0_seq1 (1022)	G / A	0.86 / 0.14	0.96 / 0.04	0.93 / 0.07	1 / 0
comp36690_c1_seq1 (361)	G / A	0.92 / 0.08	0.7 / 0.3	0.77 / 0.23	0.73 / 0.27
comp36863_c0_seq1 (1165)	G / A	0.53 / 0.47	0.32 / 0.68	0.38 / 0.62	0.11 / 0.89

comp37364_c0_seq1 (613)	G / T	0.31 / 0.69	0.29 / 0.71	0.29 / 0.71	0.01 / 0.99
comp37406_c0_seq1 (254)	G / A	0.16 / 0.84	0.58 / 0.42	0.45 / 0.55	0.96 / 0.04
comp37651_c0_seq1 (1062)	T / C	0.9 / 0.1	1 / 0	0.97 / 0.03	1 / 0
comp38584_c0_seq1 (1237)	T / C	0.27 / 0.73	0.55 / 0.45	0.47 / 0.53	0.79 / 0.21
comp38629_c0_seq1 (349)	C / G	0.89 / 0.11	0.81 / 0.19	0.83 / 0.17	0.99 / 0.01
comp38943_c0_seq1 (866)	A / G	0.12 / 0.88	0.07 / 0.93	0.08 / 0.92	0.12 / 0.88
**comp39941_c0_seq1 (1816)**	**G / A**	**0.17 / 0.83**	**0.39 / 0.61**	**0.32 / 0.68**	**0.44 / 0.56**
comp40569_c0_seq1 (764)	T / C	0.94 / 0.06	0.6 / 0.4	0.7 / 0.3	0.96 / 0.04
comp41803_c0_seq1 (1452)	C / T	0.18 / 0.82	0.08 / 0.92	0.11 / 0.89	0 / 1
**comp41821_c0_seq1 (413)**	**C / T**	**0.77 / 0.23**	**0.94 / 0.06**	**0.89 / 0.11**	**1 / 0**
comp423545_c0_seq1 (183)	C / T	0.28 / 0.72	0.1 / 0.9	0.15 / 0.85	0 / 1
comp42606_c0_seq1 (1061)	G / T	0.27 / 0.73	0.23 / 0.77	0.24 / 0.76	0.01 / 0.99
comp42726_c0_seq1 (307)	G / A	0.64 / 0.36	0.28 / 0.72	0.38 / 0.62	0.05 / 0.95
comp42805_c1_seq1 (2699)	A / G	0.34 / 0.66	0.54 / 0.46	0.49 / 0.51	0.98 / 0.02
comp42950_c0_seq1 (425)	T / C	0.97 / 0.03	0.98 / 0.02	0.98 / 0.02	0.88 / 0.12
comp43820_c0_seq1 (103)	C / G	0.75 / 0.25	0.91 / 0.09	0.87 / 0.13	1 / 0
comp44465_c0_seq1 (1110)	A / G	0.83 / 0.17	0.74 / 0.26	0.76 / 0.24	0.99 / 0.01
**comp44835_c0_seq1 (339)**	**C / G**	**0.91 / 0.09**	**0.54 / 0.46**	**0.65 / 0.35**	**0.01 / 0.99**
comp44981_c0_seq1 (281)	C / T	0.7 / 0.3	0.43 / 0.57	0.51 / 0.49	0.58 / 0.42
comp45024_c0_seq1 (839)	T / C	0.02 / 0.98	0 / 1	0.01 / 0.99	0.02 / 0.98
comp45463_c0_seq1 (2507)	G / A	0.92 / 0.08	0.82 / 0.18	0.85 / 0.15	0.77 / 0.23
comp45510_c0_seq1 (218)	G / C	0.84 / 0.16	0.46 / 0.54	0.58 / 0.42	0.24 / 0.76
comp45924_c0_seq1 (434)	T / C	0.31 / 0.69	0.41 / 0.59	0.38 / 0.62	0.01 / 0.99
comp46344_c0_seq1 (1108)	C / T	0.04 / 0.96	0.03 / 0.97	0.03 / 0.97	0.2 / 0.8
comp47007_c0_seq1 (1300)	G / T	0.49 / 0.51	0.22 / 0.78	0.3 / 0.7	0.52 / 0.48
comp47328_c0_seq1 (518)	G / T	0.38 / 0.63	0.58 / 0.42	0.52 / 0.48	0.6 / 0.4
comp47467_c0_seq1 (1161)	A / G	0.38 / 0.63	0.61 / 0.39	0.54 / 0.46	0.6 / 0.4
comp47663_c0_seq1 (1253)	G / C	0.37 / 0.63	0.39 / 0.61	0.39 / 0.61	0.01 / 0.99
comp48051_c0_seq1 (220)	C / T	0.86 / 0.14	0.7 / 0.3	0.75 / 0.25	0.59 / 0.41
comp48330_c0_seq1 (140)	T / C	0.44 / 0.56	0.38 / 0.62	0.4 / 0.6	0.03 / 0.97
comp48723_c0_seq2 (83)	G / T	0.13 / 0.87	0.22 / 0.78	0.19 / 0.81	0.99 / 0.01
comp48942_c0_seq1 (1943)	T / C	0.94 / 0.06	0.87 / 0.13	0.89 / 0.11	0.81 / 0.19
comp49679_c0_seq1 (4572)	A / G	0.94 / 0.06	0.87 / 0.13	0.89 / 0.11	0.98 / 0.02
comp49745_c0_seq1 (703)	G / C	0.19 / 0.81	0.2 / 0.8	0.19 / 0.81	0.01 / 0.99
comp49772_c0_seq1 (1003)	A / G	0.72 / 0.28	0.7 / 0.3	0.71 / 0.29	0.41 / 0.59
comp50296_c0_seq1 (1883)	T / C	0.78 / 0.22	0.68 / 0.32	0.71 / 0.29	0.76 / 0.24

comp50473_c0_seq1 (669)	A / C	0.06 / 0.94	0.07 / 0.93	0.07 / 0.93	0.03 / 0.97
**comp50552_c0_seq1 (1098)**	**A / G**	**0.68 / 0.32**	**0.57 / 0.43**	**0.6 / 0.4**	**0.4 / 0.6**
comp50851_c0_seq1 (996)	A / G	0.11 / 0.89	0.5 / 0.5	0.39 / 0.61	0.98 / 0.02
comp50905_c0_seq4 (942)	A / G	0.05 / 0.95	0.14 / 0.86	0.12 / 0.88	0.02 / 0.98
comp51215_c0_seq1 (1350)	C / G	0.72 / 0.28	0.83 / 0.17	0.8 / 0.2	0.99 / 0.01
comp51336_c0_seq1 (1958)	T / G	0.16 / 0.84	0.23 / 0.77	0.21 / 0.79	0.34 / 0.66
comp51669_c0_seq1 (313)	C / T	0.23 / 0.77	0.16 / 0.84	0.18 / 0.82	0.21 / 0.79
comp51783_c0_seq1 (508)	T / G	0.81 / 0.19	0.64 / 0.36	0.69 / 0.31	0.25 / 0.75
comp52585_c0_seq1 (127)	C / G	0.77 / 0.23	0.84 / 0.16	0.82 / 0.18	1 / 0
comp52700_c0_seq1 (1559)	C / T	0.85 / 0.15	0.84 / 0.16	0.85 / 0.15	0.84 / 0.16
**comp52994_c0_seq1 (245)**	**T / C**	**0.12 / 0.88**	**0.3 / 0.7**	**0.24 / 0.76**	**0.9 / 0.1**
comp53206_c1_seq1 (1087)	C / T	0.96 / 0.04	0.8 / 0.2	0.85 / 0.15	0.83 / 0.17
comp53528_c0_seq1 (1313)	G / A	0.97 / 0.03	0.96 / 0.04	0.96 / 0.04	0.87 / 0.13
comp53591_c0_seq1 (320)	G / A	0.13 / 0.87	0.13 / 0.87	0.13 / 0.87	0.14 / 0.86
**comp53610_c0_seq1 (276)**	**A / G**	**0.31 / 0.69**	**0.39 / 0.61**	**0.37 / 0.63**	**0.51 / 0.49**
comp53749_c0_seq7 (2413)	T / C	0.66 / 0.34	0.69 / 0.31	0.68 / 0.32	0.98 / 0.02
comp53870_c0_seq1 (109)	G / A	0.65 / 0.35	1 / 0	0.91 / 0.09	0.99 / 0.01
comp54118_c0_seq1 (2585)	A / C	0.71 / 0.29	0.47 / 0.53	0.54 / 0.46	0.3 / 0.7
**comp54487_c0_seq1 (3338)**	**T / C**	**0.18 / 0.82**	**0.26 / 0.74**	**0.24 / 0.76**	**0.01 / 0.99**
comp55083_c0_seq1 (1074)	C / T	0.72 / 0.28	0.55 / 0.45	0.6 / 0.4	0.99 / 0.01
comp56793_c0_seq1 (670)	T / G	1 / 0	0.98 / 0.02	0.98 / 0.02	0.86 / 0.14
comp57453_c0_seq1 (2049)	A / G	0.25 / 0.75	0.3 / 0.7	0.29 / 0.71	0.01 / 0.99
comp58612_c0_seq1 (681)	A / G	0.81 / 0.19	0.92 / 0.08	0.89 / 0.11	0.99 / 0.01
comp59746_c0_seq1 (1253)	T / G	0.78 / 0.22	0.64 / 0.36	0.68 / 0.32	0.78 / 0.22
comp59967_c0_seq1 (1910)	G / A	0.2 / 0.8	0.28 / 0.72	0.25 / 0.75	0.23 / 0.77
comp60629_c0_seq1 (1021)	T / C	0.64 / 0.36	0.83 / 0.17	0.78 / 0.22	0.99 / 0.01
comp61109_c0_seq1 (1237)	C / A	0.87 / 0.13	0.97 / 0.03	0.94 / 0.06	0.96 / 0.04
comp63308_c0_seq1 (1097)	C / T	0.82 / 0.18	0.7 / 0.3	0.74 / 0.26	0.85 / 0.15
comp64225_c0_seq1 (1194)	G / T	0.45 / 0.55	0.23 / 0.77	0.29 / 0.71	0.01 / 0.99
comp71632_c0_seq1 (1208)	C / T	0.03 / 0.97	0.09 / 0.91	0.07 / 0.93	0.09 / 0.91
comp72132_c0_seq1 (584)	A / G	0.89 / 0.11	0.93 / 0.07	0.92 / 0.08	0.93 / 0.08
comp74829_c0_seq1 (165)	C / A	0 / 1	0.1 / 0.9	0.07 / 0.93	0.04 / 0.96
comp1178_c0_seq1 (146)	G / A	0.01 / 0.99	0.03 / 0.97	0.02 / 0.98	0.09 / 0.91
comp166107_c0_seq1 (81)	A / G	0.75 / 0.25	0.54 / 0.46	0.6 / 0.4	0.13 / 0.87
comp19558_c0_seq1 (688)	A / G	0.87 / 0.13	0.89 / 0.11	0.89 / 0.11	0.99 / 0.01
comp21559_c0_seq1 (165)	T / C	0.28 / 0.72	0.1 / 0.9	0.15 / 0.85	0 / 1

comp227564_c0_seq1 (556)	A / G	0.96 / 0.04	0.77 / 0.23	0.83 / 0.17	0.48 / 0.52
comp29071_c0_seq1 (277)	T / G	0.53 / 0.47	0.35 / 0.65	0.4 / 0.6	0.07 / 0.93
comp37643_c0_seq1 (1161)	G / A	0.59 / 0.41	0.25 / 0.75	0.34 / 0.66	0.01 / 0.99
comp39687_c0_seq1 (706)	C / T	0.25 / 0.75	0.23 / 0.78	0.23 / 0.77	0.2 / 0.8
comp40831_c0_seq1 (622)	C / T	0.18 / 0.82	0.33 / 0.67	0.29 / 0.71	0.45 / 0.55
comp43127_c0_seq1 (312)	A / C	0.36 / 0.64	0.06 / 0.94	0.15 / 0.85	0.21 / 0.79
comp45714_c0_seq1 (1064)	C / A	0.56 / 0.44	0.84 / 0.16	0.75 / 0.25	1 / 0
comp48574_c0_seq1 (1639)	G / A	0.57 / 0.43	0.6 / 0.4	0.59 / 0.41	0.01 / 0.99
comp49514_c0_seq1 (63)	A / G	0.3 / 0.7	0.11 / 0.89	0.17 / 0.83	0.25 / 0.75
comp49840_c0_seq1 (2135)	C / G	0.72 / 0.28	0.81 / 0.19	0.79 / 0.21	0.97 / 0.03
comp51626_c0_seq1 (1245)	C / T	0.59 / 0.41	0.83 / 0.17	0.76 / 0.24	0.91 / 0.09
comp52601_c0_seq1 (205)	A / G	0.28 / 0.72	0.06 / 0.94	0.13 / 0.87	0.07 / 0.93
comp58030_c0_seq1 (550)	A / G	0.08 / 0.92	0.34 / 0.66	0.26 / 0.74	0.2 / 0.8
comp66672_c0_seq1 (661)	C / G	0.86 / 0.14	0.62 / 0.38	0.69 / 0.31	0.29 / 0.71
0_4394_01_1 (522)	G / A	0.39 / 0.61	0.28 / 0.72	0.32 / 0.68	0.01 / 0.99
CL12234_1_1 (626)	A / G	0.01 / 0.99	0.01 / 0.99	0.01 / 0.99	0.33 / 0.67

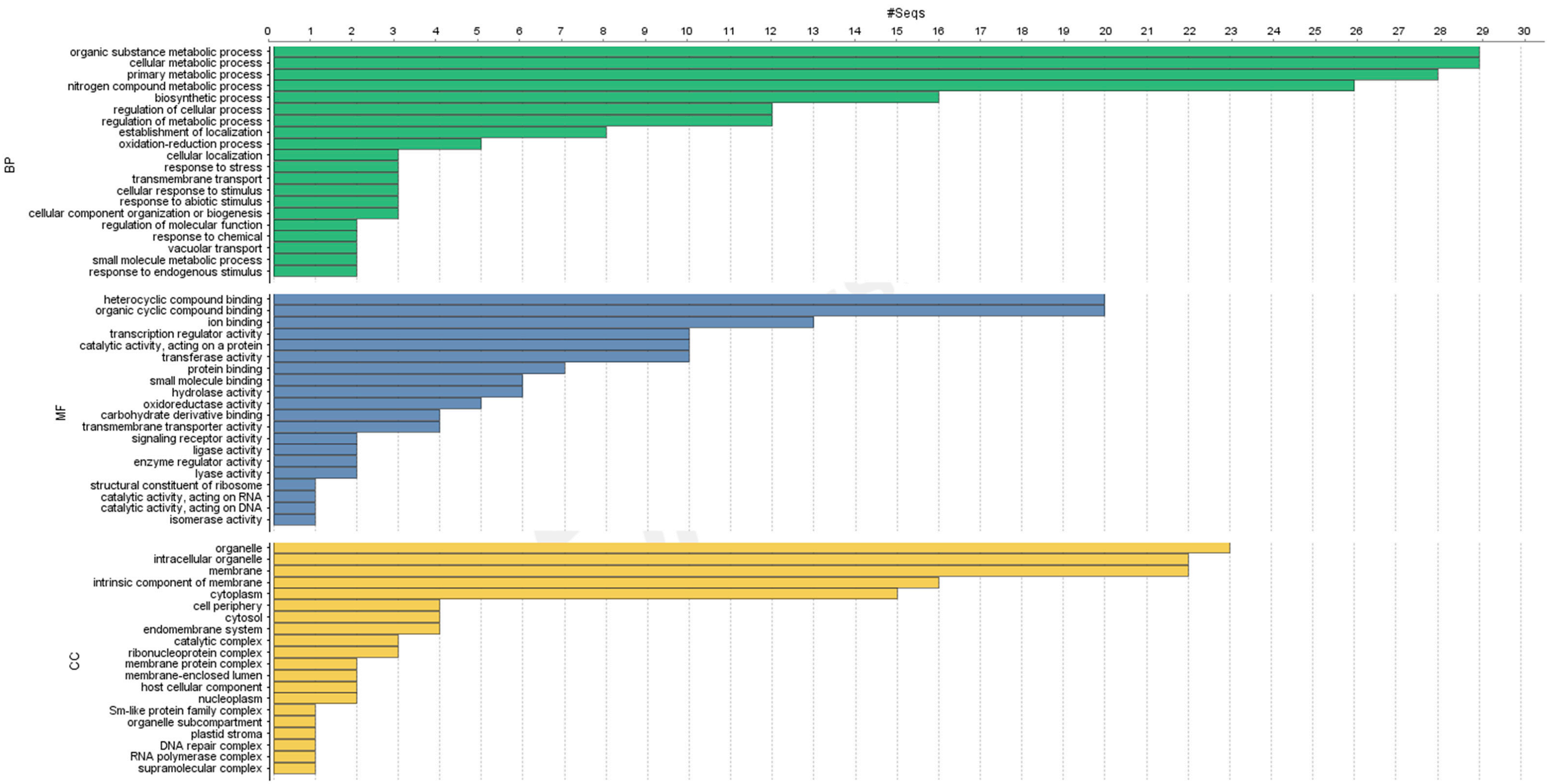

Figure S7. Distribution of top 20 third-level gene ontologies in each of the three main GO categories annotated for 64 sequences in which outlier SNPs were detected between dwarf and Pyrenean pines; BP - Biological Processes (annotated for 48 sequences), MF - Molecular Functions (50 sequences), CC - Cellular Components (38 sequences).
